# Co-evolution of behaviour and beliefs in social dilemmas: estimating material, social, cognitive and cultural determinants

**DOI:** 10.1017/ehs.2024.38

**Published:** 2024-12-03

**Authors:** Sergey Gavrilets, Denis Tverskoi, Nianyi Wang, Xiaomin Wang, Juan Ozaita, Boyu Zhang, Angel Sánchez, Giulia Andrighetto

**Affiliations:** 1 Department of Ecology and Evolutionary Biology, Department of Mathematics, University of Tennessee, Knoxville, TN 37996, USA; 2 National Institute for Mathematical and Biological Synthesis, University of Tennessee, Knoxville, TN 37996, USA; 3 Health and Environment Modeling Laboratory, The Ohio State University, Columbus, OH 43210, USA; 4 Laboratory of Mathematics and Complex Systems, Ministry of Education, School of Mathematical Sciences, Beijing Normal University, Beijing 100875, People's Republic of China; 5 Grupo Interdisciplinar de Sistemas Complejos, Departamento de Matemáticas, Universidad Carlos III de Madrid, 28911 Leganés, Madrid, Spain; 6 Instituto de Biocomputación y Física de Sistemas Complejos, Universidad de Zaragoza, 50018, Zaragoza, Spain; 7 Institute of Cognitive Sciences and Technologies, Italian National Research Council, Rome, Italy; 8 Institute for Futures Studies, Stockholm, Sweden; 9 Institute for Analytical Sociology, Linkoping University, Sweden

**Keywords:** Behavioral experiments, social norms, cognitive processes, rule-following, prosociality, cultural variation

## Abstract

Understanding and predicting human cooperative behaviour and belief dynamics remains a major challenge both from the scientific and practical perspectives. Because of the complexity and multiplicity of material, social and cognitive factors involved, both empirical and theoretical work tends to focus only on some snippets of the puzzle. Recently, a mathematical theory has been proposed that integrates material, social and cognitive aspects of behaviour and beliefs dynamics to explain how people make decisions in social dilemmas within heterogeneous groups. Here we apply this theory in two countries, China and Spain, through four long-term behavioural experiments utilising the Common Pool Resources game and the Collective Risk game. Our results show that material considerations carry the smallest weight in decision-making, while personal norms tend to be the most important factor. Empirical and normative expectations have intermediate weight in decision-making. Cognitive dissonance, social projection, logic constraints and cultural background play important roles in both decision-making and beliefs dynamics. At the individual level, we observe differences in the weights that people assign to factors involved in the decision-making and belief updating process. We identify different types of prosociality and rule-following associated with cultural differences, various channels for the effects of messaging, and culturally dependent interactions between sensitivity to messaging and conformity. Our results can put policy and information design on firmer ground, highlighting the need for interventions tailored to the situation at hand and to individual characteristics. Overall, this work demonstrates the theoretical and practical power of the theory in providing a more comprehensive understanding of human behaviour and beliefs.

## Graphical abstract

**Figure:**
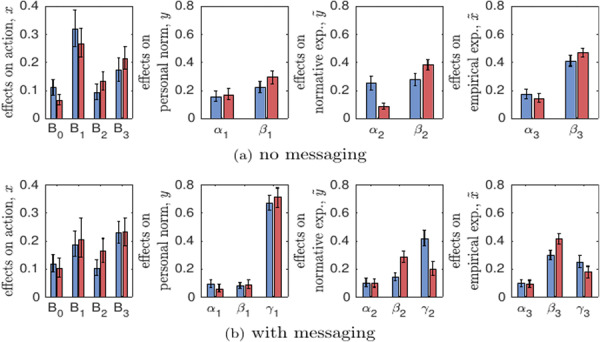

**Social media summary:** Experiments measure material, social, cognitive and cultural effects on behaviour and belief dynamics in social dilemmas

## Introduction

Our society grapples with numerous challenges including climate change, pandemics, inequality, economic crises, political polarisation, misinformation, violent conflicts and refugee crises. Solving these requires consensus-based policies, adequate funding, technological capabilities and a deep understanding of human behaviour and beliefs. The challenge with the latter lies in the multitude of factors shaping individual preferences and decision-making, along with the intricate mutual influences within social networks which make human groups very complex coevolving systems. This complexity is illustrated by a highly cited review published in 2015 which listed as many as 82 different theories of behaviour and behavioural change (Davis et al., 2015).

Attempts to develop a much needed integrative theory of behaviour and beliefs dynamics have resorted to two, largely independent, general approaches. One is various flavours of non-cooperative game theory (Fudenberg & Tirole, 1992; Sandholm, 2010; Tembine, 2017; Piotrowski & Sladkowski, 2003), centred on strategic interactions and actions maximising individual payoffs or utility. The second is different social influence models describing how individuals change actions, beliefs or preferences upon obtaining information about the behaviour and/or beliefs of others (Rashevsky, 1949; DeGroot, 1974; Granovetter, 1978; Cavalli-Sforza & Feldman, 1981; Boyd & Richerson, 1985; Watts, 2002; Jackson, 2010; Easley and Kleinberg, 2010). Middle ground theoretical models include beliefs into utility functions used in game theoretic models (Akerlof, 1980; Akerlof & Dickens, 1982; Kuran, 1989; Rabin, 1994; Geanakoplos et al., 1989a; Battigalli & Dufwenberg, 2022), introduce payoff-biased imitation in models of social influence and cultural evolution (Boyd & Richerson, 1985; Sandholm, 2010) or add learning dynamics based on the actions of others (Camerer, 2003). While leading to important results and insights, these attempts towards an integration are incomplete as the crucial role of belief dynamics in social norms, i.e. shared beliefs about what should or should not be done (Cialdini & Goldstein, 2004; Bicchieri, 2006), is often overlooked. This calls for an integrated theory that could, first, explicitly include the effect of beliefs in the decision-making process, and second, describe accurately the dynamics of the beliefs as controlled by the decisions taken and observed (Loewenstein & Molnar, 2018; Molnar & Loewenstein, 2022; Galesic et al., 2021; Gavrilets et al., 2024). In other words, there is a need for a theory that properly accounts for the two-way feedback loop between behaviour and beliefs that is at the heart of human behaviour.

Decision-making and beliefs dynamics are also affected by psychological factors that may not be directly related to material payoffs or social influence. Examples include internalised norms (Schwartz, 1977; Henrich & Ensminger, 2014; Catola et al., 2021), cognitive dissonance (Festinger, 1957), theory of mind (Premack & Woodruff, 1979; Baron-Cohen et al., 1985), social projection (Krueger, 2007) and logic constraints (Friedkin et al., 2016; Rawlings, 2020). Attempts have been made to include these into game-theoretic (Geanakoplos et al., 1989b; Rabin, 1994; Calabuig et al., 2018; Battigalli & Dufwenberg, 2022) and social influence models (Friedkin et al., 2016), but the need for a better integration of cognitive processes with models of human behaviour and beliefs dynamics remains (Galesic et al., 2021; Gavrilets et al., 2024). Moreover, understanding social interactions requires accounting for between-individual variation (Gavrilets 2015). While differences in actions/strategies, opinions/beliefs or social network structure have been considered in existing models, they usually ignore differences in physical, morphological, psychological and cognitive characteristics directly affecting both decisions and beliefs.

Recently, a new mathematical framework, inspired by behavioural experiments (d'Adda et al., 2020; Andreozzi et al., 2020; Górges & Nosenzo, 2020; Szekely et al., 2021; Gächter et al., 2021), was introduced by Gavrilets (2021). This framework investigates how individual actions in social dilemmas interact with three core types of beliefs commonly studied in social psychology: personal norms, normative expectations and empirical expectations. Personal (internalised) norms are internal standards and rules that individuals feel obligated to follow (Wrong, 1961; Campbell, 1964; Schwartz, 1977; Etzioni, 2000; Cooter, 2000; Henrich & Ensminger, 2014). These norms are self-imposed and represent the individual's perception of what behaviours are appropriate or necessary in certain situations. Personal norms develop from a blend of social, psychological and cultural factors. For instance, they may arise through learning (Gintis, 2003), by internalising social norms (Gavrilets & Richerson, 2017) or via fitness maximisation processes involving genetic relatedness (Alger & Weibull, 2013; Akçy & Cleve, 2021). Normative expectations and empirical expectations are beliefs about what others believe is right and what others are likely to do, respectively (Bicchieri, 2006). These notions are closely related to the notions of descriptive and injunctive social norms (Cialdini et al., 1990). Normative expectations can be viewed as individual perceptions of personal norms of others (Tremewan & Vostroknutov, 2021). The framework accounts for the effects of a combination of well-understood material, social and cognitive forces, and is described using simple dynamic equations amenable to statistical analysis. Subsequently, the theory was applied to energy saving behaviour (Tverskoi et al., 2021), the spread of technological innovations (Tverskoi et al., 2022), the effects of inequality between identity groups on social unrest (Houle et al., 2022; Rosokha et al., 2024) and the effects of inculcation, propaganda and social identity on cooperation (Gavrilets & Richerson, 2022).

This theoretical framework underwent validation and parameterisation through a Common Pool Resources (CPR) behavioural experiment, with and without messaging aimed at promoting group-beneficial resource extraction (Tverskoi et al., 2023). We used the CPR game because it is considered much more realistic than alternative social dilemma games (Ostrom et al., 1992). Additionally, the CPR game features an internal Nash equilibrium (a Nash equilibrium within the strategy space and not on the boundary), which is expected to lead to more diverse behaviours among subjects. Furthermore, it was predicted to exhibit backfiring effects in response to messaging (Gavrilets, 2021). We used multi-day online experiments (one round per day) because they allowed us to better observe the emergence and evolution of social norms (Szekely et al., 2021). Additionally, the multi-day setup has proven effective in preventing attrition, as participants can make their daily decisions within an almost 24 hour window, avoiding the need to spend substantial contiguous time in the experiment (see below for details). Previous experimental work has demonstrated the existence of distinct types of individuals with varying behaviours in social dilemmas, significantly impacting group dynamics (Fischbacher & Gächter, 2010; Andreozzi et al., 2020; Szekely et al., 2021). Building on this research, we conducted a detailed examination of inter-individual differences using results from the Social Value Orientation test (Murphy et al., 2011), rule-following tests (Kimbrough & Vostroknutov, 2016, 2018), cluster analysis and additional analyses of individual behaviours. This comprehensive approach allowed us to identify specific classes of individuals, such as stubborn individuals and conditional compliers.

The findings revealed intriguing insights, highlighting the dominant influences on decision-making and belief dynamics. Notably, personal norms and conformity to expected peer behaviour emerged as the most significant factors, whereas material benefits and normative expectations exerted relatively smaller effects. Prosocial individuals exhibited stronger adherence to personal norms, while antisocial tendencies were more influenced by conformity. The introduction of messaging led to a reduction in the weight of personal norms and a concurrent increase in conformity, alongside noticeable alterations in personal norms and normative expectations. The dynamics of beliefs were found to be shaped by both cognitive and social factors, with interindividual variability significantly impacting group behaviour outcomes. Overall, the results underscored the indispensability of comprehending the interplay of personal beliefs, the perceptions of others and the intricate roles played by cognitive, social and material factors in shaping social behaviour.

While the findings from the exploratory study by Tverskoi et al. (2023) were interesting, their generalisability remained uncertain owing to the focus on a single behavioural game and a single pool of subjects from Spain. In this study, we present the results of a similar experiment conducted with Chinese participants. By including both Western and non-Western subject pools, we aim to investigate the influence of cultural differences on human behaviour and belief dynamics (Henrich et al., 2010; Henrich, 2020; Muthukrishna et al., 2020). Specifically, we seek to understand how the expected higher conformity and cultural tightness among Chinese participants (Gelfand et al., 2011) impact the observed dynamics of key variables and parameter estimates. Additionally, we employ our theoretical framework to analyse results from two published Collective Risk (CR) experiments (Szekely et al., 2021; Vriens et al., 2024). We chose this game owing to its significant potential in understanding methods to mitigate ongoing climate change. Additionally, previous experiments by Szekely et al. (2021) and Vriens et al. (2024) collected the exact data needed to fit our model, making this game particularly suitable for our research. Unlike the CPR game, it provides clearer expectations for participants (specifically, contributing a fair share to avoid a collective catastrophe), which suggests a stronger influence of social norms.

This stark difference prompts us to look deeper into the dynamics of decision-making and belief evolution across these two experimental setups. Our comparative analysis between the outcomes of the CPR and CR experiments seeks to not only affirm but also refine our understanding of the relative strengths of various influential factors in shaping individual behaviours and belief dynamics.

In the next section, we outline our general research framework, beginning with a description of the dynamic mathematical model and followed by an experimental setup. We then proceed to discuss the results of the CPR and CR experiments, initially examining them separately before drawing comparisons. Finally, we offer a summary of our findings and engage in a comprehensive discussion to elucidate their broader implications.

## General approach

### Modelling framework

Consider individuals interacting in groups. Let us designate an action chosen by a specific individual by a continuous variable *x*. Each individual possesses an attitude *y*, reflecting their perception of the most suitable action in a given social circumstance. They also hold a first-order belief or prediction (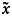) regarding their peers’ average action, along with a second-order belief (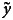) about their peers’ average attitude. Adopting terms from social psychology, we refer to *y*, 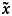, and 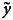 as a personal norm, empirical expectation and normative expectation, respectively (Schwartz, 1977; Cialdini et al., 1990; Bicchieri, 2006; Szekely et al., 2021). The empirical expectation 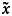 can be viewed as a descriptive norm (representing the most frequent behaviour), while the normative expectation 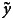 can be interpreted as an injunctive norm (signifying socially appropriate behaviour), both as understood by the individual (Cialdini et al., 1990; Bicchieri, 2006; Gavrilets, 2020). Furthermore, we assume individuals are susceptible to influence from an external authority advocating for a specific action *G*. We postulate that *x*, *y*, 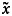, *y*, *G* are non-negative.

Gavrilets’ (2021) modelling framework predicts the following relationship between the action *x* the individual chooses and variables *y*, 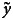, 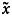 and *G*:1
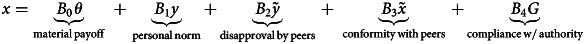
where *θ* is the action maximising the expected material payoff *π*(*x*, 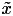) (Gavrilets, 2021; Tverskoi et al., 2023). For the payoff functions used below, *θ* can be found in a straightforward way (see SM). Coefficients *B_i_* are the relative weights of material factors, personal norms, normative expectations, empirical expectations, and messaging in the decision made, respectively (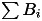).

After taking actions and observing behaviour of groupmates, the attitude and beliefs of a focal individual change. We describe these changes using linear recurrence equations:2*a*
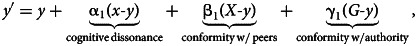
2*b*
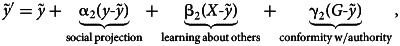
2*c*
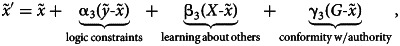
where the prime means the next time step, *X* is the average action of groupmates as observed by the focal individual (so that different individuals can have different *X*), and *α_i_*, *β_i_*, *γ_i_* are non-negative constant coefficients measuring the strength of the corresponding forces. Here the ‘cognitive dissonance’ term acts to reduce the mismatch of the ego's action and their belief about the right behaviour. The ‘social projection’ term captures the ego's belief that others are probably similar to themselves (Premack & Woodruff, 1979; Krueger, 2007). The ‘logic constraints’ term reduces the mismatch between the ego's beliefs about actions and beliefs of others (cf. Friedkin et al., 2016). The ‘conformity w/ peers’ and the two ‘learning about others’ terms move the corresponding beliefs closer to the observed average behaviour *X* of peers (Fischbacher & Gächter, 2010; Kashima et al., 2015). The ‘conformity w/ authority’ terms move the corresponding beliefs closer to the promoted ‘standard’ *G*. Note that cognitive dissonance makes individuals to choose an action *x* closer to their attitude *y* (as implied by equation 1) and simultaneously changes their attitude *y* to justify the action previously chosen (as described by the first term in equation 2a; cf. Rabin, 1994). The authority's messaging simultaneously affects actions (equation 1) and beliefs (equation 2) which then feed back into behaviour. All parameters defined above are individual specific; we estimate their average values using experimental data.

### Two games

We applied the above framework to two social dilemmas.

#### Common Pool Resources game

In this game, individuals in a group of size *n* make efforts *x_i_* to extract resources from a common pool (Walker et al., 1990; Ostrom et al., 1992; Apesteguia, 2006; Apesteguia & Maier-Rigaud, 2006). The total group effort 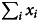 defines the amount *P*(*X*) of resources extracted by the group. The share of the resource going to individual *i* is proportional to their effort: *x_i_*/*X*. It is assumed that the production function *P*(*X*) is characterised by diminishing return: *P*(*X*) = *bX* −0.5*dX*^2^, where *b* and *d* are positive constant parameters. In this model, an individual payoff function is3
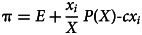


where *c* is a constant cost coefficient and *E* is a personal endowment. Standard game-theoretic analysis shows that in the case of perfect rationality, there is a unique Nash equilibrium
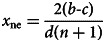
while the level of extraction maximising the total group benefit is
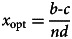
i.e. the Nash equilibrium value is 2*n/*(*n*+1) times larger than the socially optimal value (e.g. Gavrilets, 2021; Tverskoi et al., 2023).

#### Collective Risk game

In this game, a group of *n* individuals are facing a risk of losing their personal endowments of size *E* in the event of a disaster which happens with a fixed probability *p* (see Milinski et al., 2008; Szekely et al., 2021). Individuals can prevent the disaster by making contributions *x_i_* to a joint pool if their total contribution 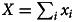 matches or exceeds a certain threshold *X*_0_. If the disaster does happen, the payoff to each individual is zero: *π_i_* = 0. If the disaster does not happen, the individual payoff is whatever is left after making a contribution to the joint pool: *π_i_* = *E* −*x_i_*. It is assumed that *nE > X*_0_, so that it is possible to prevent the disaster. These assumptions lead to the expected individual payoff:4
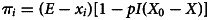
where *I*(*z*) is the Heaviside function equal to 0 if *z* ≤ 0 and to 1 if *z >* 0.

In this game there are two symmetric Nash equilibria (at which all individuals make the same contribution). At one equilibrium, individuals make zero contributions, *x*_ne_ = 0. At the other equilibrium, each individual contributes a fair share: *x*_ne_ = *X*_0_*/n* and the disaster is always prevented. The second equilibrium ensures a higher expected payoff if the probability of disaster *p* is larger than the critical value
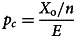


There is also a continuum of asymmetric Nash equilibria at which individuals make different contributions. At these equilibria, the total group effort *X* matches *X*_0_ exactly and the disaster is always prevented.

## Experimental approach

All experiments used the same experimental protocol as described in detail in Szekely et al. (2021) and Tverskoi et al. (2023). For each game and treatment, in each round of the experiment, conducted daily, 150 participants were randomly re-assigned to groups of six. They were then given an endowment to engage in the game.

The collected data represented individual actions *x* (i.e. the amount each participant chose to utilise in the game), subjects’ personal norms *y*, normative expectations 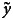 and empirical expectation 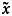 for each round. Table 1 specifies how these variables were measured. The questions about empirical and normative expectations were incentivised so that subjects received extra payments based on the accuracy of their answers (Bicchieri, 2006; Gächter & Renner, 2010; Szekely et al., 2021; and Tverskoi et al., 2023: 11). In the Common Pool Resource games, participants made decisions subsequent to the elicitation of their beliefs, while in the Collective Risk games the sequence of assessing actions and beliefs was randomised. In the latter, no statistically significant differences were observed in the actions taken when beliefs were elicited before or after the action (Szekely et al., 2021). After each round, subjects were informed about their own payoffs and the actions taken by their groupmates. The Common Pool Resources experiments lasted 35 rounds (one round per day), whereas the Collective Risk experiments spanned 28 rounds.

**Table 1.** Main variables measured in the experiments each round for each individual

Name	Notation	Questions used to elicit actions and beliefs in all experiments
Action (extraction/contribution)	*x*	How many points will you extract/contribute?
Personal norm	*y*	How many points should a person in your group extract/contribute?
Empirical expectation	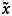	How many points did/will the other five people in your group extract/contribute?
Normative expectation	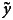	How many points did/will the other five people in your group think you should extract/contribute?

Excluding the additional payment from the lottery, subjects in the CPR games earned an average an equivalent of Euro 31.65; in the CR games, this amount was about Euro 21. The lottery was introduced to keep subjects engaged until the end of the experiment. Specifically, in the CPR games three participants were randomly selected from those who have completed all parts of the experiment to receive an additional payment, consisting of a 10-fold increase in their earnings. In the CR games, two participants were selected to receive a flat payment of Euro 100 additional to their earnings. A few subjects dropped out or were excluded (see Table S4.2 for the sample sizes used), with the attrition rate being less than 10% in all experiments. The experiment was coded in oTree (Chen et al., 2016). Every day at 10:00 a.m. participants received a link to participate in either the corresponding game and in the beliefs elicitation task. They had 24 hours to make their decisions for that day.

In each experiment, participants also completed the Social Value Orientation (SVO) test (Murphy et al., 2011; Murphy & Ackermann, 2014) and the Rule-Following test (Kimbrough & Vostroknutov, 2018; see Sections S3.3 and S3.4 of the Supplementary Material, SM). The SVO test categorised participants into prosocial and individualistic types, while the Rule-Following test categorised them into rule-followers and rule-breakers. Both social value orientation (Ackermann & Murphy, 2019) and rule-following tendencies (Kimbrough & Vostroknutov, 2018) have been identified as significant predictors of behaviour in social dilemmas. Contrasting the behaviours of these types in our experiments and the corresponding parameter estimates provides additional independent tests of the consistency of our approach. Moreover, it allows us to look deeper into the differences between prosocial and individualistic types, as well as between rule-followers and rule-breakers, in terms of various social, cognitive, and cultural factors.

Our mathematical model explicitly describes the dynamics of actions *x* and beliefs *y*, 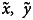 (equations 1 and 2, and Table 1). Our model-based analysis has allowed us to measure different forces (material, cognitive, social and cultural) driving behaviour and beliefs on exactly the same scale in the same experimental setup. It has also allowed us to uncover some interactions between these forces. Table 2 summarises the parameters estimated by our method. The results presented below focus on these parameters and on observed dynamics of *x*, *y*, 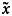, 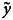 comparing them across the cultural background of subjects, economic games used and the treatments applied.

**Table 2.** Estimated parameters of the model measuring the weights of corresponding factors in decision-making and beliefs dynamics

Type	Notation	Underlying factors
Decision-making parameters	*B*_0_	Material payoff
	*B*_1_	Personal norm (cognitive dissonance)
	*B*_2_	Normative expectation (injunctive social norm)
	*B*_3_	Empirical expectation (descriptive social norm)
Belief dynamics parameters	*α*_1_, *α*_2_, *α*_3_	Cognitive (cognitive dissonance for personal norms, social projection for normative expectations, and logic constraints for empirical expectations)
	*β*_1_, *β*_2_, *β*_3_	Observed peers’ behaviour (for personal norms, normative expectations and empirical expectations, respectively)
	*γ*_1_, *γ*_2_, *γ*_3_	Messaging (for personal norms, normative expectations and empirical expectations, respectively)

### Estimation

For statistical analysis of each experiment we used the method developed in Tverskoi et al. (2023) which should be consulted for more details. Briefly, employing the mean group estimator (Pesaran and Smith 1995), we estimated the parameters of equations (1) and (2) individually for each subject, and then averaged them across the entire group. Individual estimates for each subject were produced as follows. For each individual, we considered a set of candidate models. Each candidate model was obtained from the baseline model (described by equation 2 for actions, or by equation 3 for beliefs) by excluding a subset of explanatory variables. This results in 32 candidate models for actions and either 16 (CPR experiments with messaging) or eight (other cases) candidate models for beliefs. For each candidate model, we checked for multicollinearity (Belsley, 1991; Belsley et al., 2005) and used ridge regression (Hoerl & Kennard, 1970) if multicollinearity was identified. Otherwise, standard ordinary least squares estimates were obtained. For individual estimates, we employed model averaging (Burnham & Anderson, 2001) using the Akaike Information Criterion weights corrected for small sample sizes. Given individual estimates for each subject, mean-group estimates were obtained (Pesaran and Smith, 1995). The corresponding confidence intervals were produced employing non-parametric bootstrap analysis. With our data, we were able to estimate 13 out of the 14 parameters in our model. However, we could not estimate parameter *B*_4_. In our CPR experiments, we used a single value of *G* = 14, which means that the term *B*_4_ *G* in the best response equation (1) is a constant which cannot be differentiated from the effects of other forces represented by an intercept. Note that although in the CPR experiments we are not able to estimate the direct effects of messaging on actions, we estimate its direct effects on personal norms, normative expectations and empirical expectations. These variables in turn directly control individual actions. The treatment with messaging was absent in the CR experiments. We tested our statistical approach using agent-based simulations, which demonstrated the method's ability to recover known parameter values from simulated data and accurately describe observed mean trajectories (Section S2.5 in the SM and Tverskoi et al., 2023). For completeness and to simplify various comparisons, in the graphs shown below we include our previously published results from the CPR-Spain experiment (Tverskoi et al., 2023)

## Results

### Common pool resources experiments

Two experiments used the CPR game with two treatments: one with and one without a message indicating what is the best action for the whole group. The CPR game models the consumption of depletable, rival resources (Ostrom et al., 1992), where the individual and collective interests are in conflict and the most beneficial outcome does not align with the Nash equilibrium. Contrasting the treatments without and with messaging makes it possible to examine the effects of nudging, propaganda and backfiring (Bernays, 1928; Jowett & O'Donnell, 1992; Rozenas & Stukal, 2019; Sunstein, 2021), which are of great theoretical interest and practical importance. The first experiment, referred to as CPR-Spain, involved participants from Spain and took place in 2020. Although its findings have been previously published (Tverskoi et al., 2023), we include them here as a baseline for comparison to enrich our understanding of the results and implications of our new analyses. The second experiment, dubbed CPR-China, is new; we carried it out in 2022 with Chinese participants using exactly the same experimental protocol as in CPR-Spain (Tverskoi et al., 2023). For the instructions explaining the experiments to the participants see the SM. Using both Western and non-Western subject pools enables us to explore the impact of cultural differences on human behaviour and the dynamics of belief formation (Henrich et al., 2010; Henrich, 2020; Muthukrishna et al., 2020). In these experiments, in each round (35 rounds, one per day) participants received a 30 point endowment; a Nash equilibrium was at 24 points, while 14 points maximised group benefit. The investment in the CPR extraction was described to the subjects as a contribution to the ‘Common Account’ while the investment into a safe activity as a contribution to a ‘Personal Account’ (see the SM in Tverskoi et al., 2023). In the experiment with messaging, at each round subjects saw a message ‘Please note that the total group profit is maximised if each player contributes 14 points to the Common Account’. At each round, before making decisions, participants were asked about their personal norms and empirical and normative expectations in an incentive-compatible way.

### Trajectories

**Figure 1.**
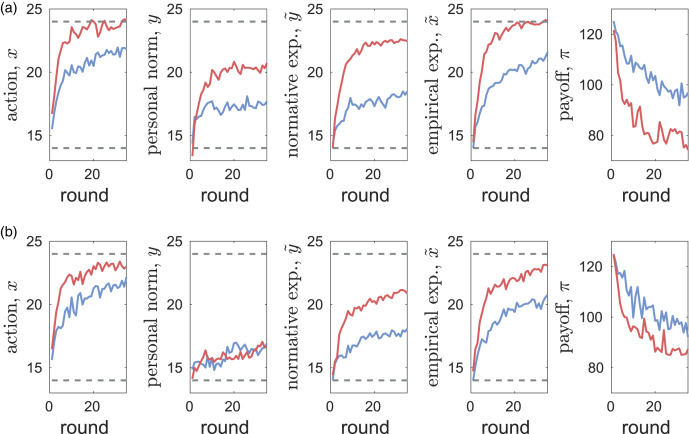
The dynamics of mean values of extraction effort *x*, personal norm *y*, normative expectation 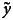, empirical expectation 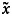 and material payoff *π* in the Common Pool Resources (CPR)-Spain (blue) and CPR-China (red) experiments for the cases without (a) and with (b) messaging. Dashed horizontal lines show the social optimal effort *x*_opt_ = 14, and the Nash equilibrium *x*_ne_ = 24.

Figure 1 shows the dynamics of the mean values of main variables *x*, *y*, 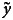, 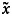 as well as the payoffs *π* in the two CPR experiments (see also Figure S2 in the SM). Some patterns are common across the experiments. The mean initial values of all variables are very similar across experiments and treatments (see Section S2.1 in the SM for details). In all cases, mean efforts *x* start at values above the socially optimal value at *x*_opt_ = 4 and appear to evolve to values below the Nash equilibrium at 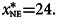 (see Figure S4 and Section S2.3 in the SM for details). In all experiments, increasing exploitation of the resource leads to a reduction in payoffs *π*. Increasing exploitation effort *x* is tracked most closely by empirical expectations 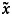. Personal norms *y* equilibrate at much smaller values than efforts *x* while the asymptotic values of normative expectations 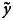 are intermediate between those of *x* and *y*. With messaging, the dynamics of average personal norms *y* are very similar in both experiments (Figure 1b) and are not too far from the value promoted by messaging (*G* = 14). Standard deviations (see Figure S2 in the SM) are the highest in actions *x*, followed by those in personal norms *y* and in normative expectations 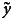 while variation in empirical expectations 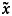 is the smallest as predicted theoretically in Gavrilets (2021). On average, personal norms tend to equilibrate the fastest while empirical expectations tend to take the longest time to equilibrate. We note that the dynamics of actions in our experiments exhibit similarities to those seen in previous CPR experiments (Walker et al., 1990; Apesteguia & Maier-Rigaud, 2006; Apesteguia, 2006), where the efficacy of best response predictions was also noted (Apesteguia, 2006).

There are also striking differences between the experiments (see Figure 1). All four main variables are higher in CPR-China than in CPR-Spain (except for personal norms in the case with messaging). Correspondingly, payoffs are lower in CPR-China. While messaging decreases all variables in CPR-China, in CPR-Spain messaging decreases personal norms *y* but has no effect on average actions *x* and only weak effects on normative and empirical expectations 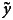 and 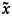 (see Figure S2 in the SM). The differences between CPR-Spain and CPR-China are larger without messaging (Figures 1a) than with it (Figure 1b). Without messaging, the personal norm *y* in CPR-China is much larger than in CPR-Spain. The differences between personal norms *y* and normative expectations 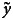 are much larger in the CPR-China than in the CPR-Spain experiments. In CPR-China, this difference is particularly large with messaging. This suggests that while Chinese subjects have the content of their own personal norm reduced by messaging (i.e. they believe a lower extraction from the common account is appropriate), they believe that personal norms of others will not be much affected. In contrast, in CPR-Spain it appears that subjects assume that others have only slightly higher personal norms than they do themselves. The dynamics of the average of personal norms *y* in CPR-Spain with messaging exhibit a step-like increase in the middle of the experiment. We discuss its causes below.

### Parameter estimates

**Figure 2.**
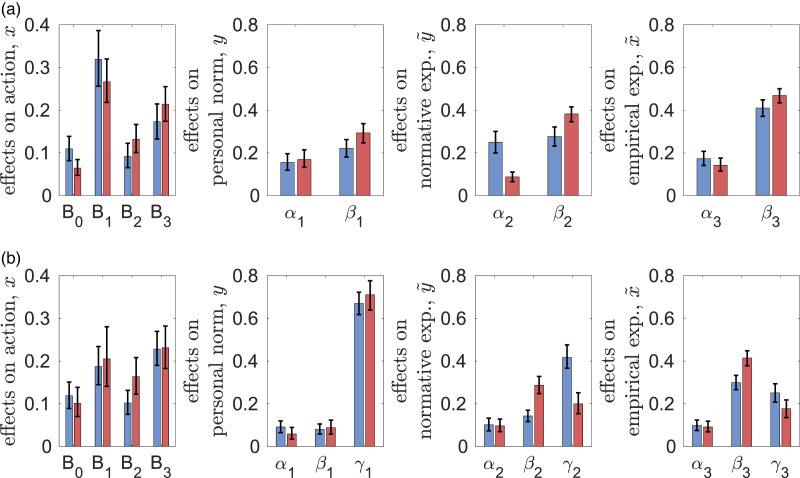
Estimates of parameters of decision-making *B*_0_, *B*_1_, *B*_2_, *B*_3_ and beliefs dynamics *α_i_*, *β_i_*, *γ_i_* (with 95% bootstrap confidence intervals) in the two experiments: CPR-Spain (blue) and CPRChina (red).

Figure 2 shows the estimates of parameters of decision-making and beliefs dynamics (explicitly defined in Materials and Methods). First, all parameters are significantly different from zero, meaning that all the corresponding effects are important for decision-making and beliefs updating. There is qualitative similarity between the experiments: personal norms and empirical expectations have the largest associated weights (*B*_1_ and *B*_3_, respectively), while material factors and normative expectations (*B*_0_ and *B*_2_, respectively) the smallest. Messaging greatly reduces the weights of cognitive factors in decision-making (parameter *B*_1_) and belief dynamics (parameters *α*_1_, *α*_2_, *α*_3_) and also causes some increase in the effect of observed behaviour of others on actions (parameter *B*_3_). For first- and second-order beliefs (*y*, 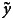, 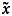), the observed behaviour of peers (parameters *β_i_*) is at least as important as cognitive factors (parameters *α_i_*). The weight of observed behaviour is the largest for empirical expectations 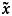. Messaging is most important for personal norms (parameter *γ*_1_), where it greatly over-weights the two other factors, and least important for empirical expectations (parameter *γ*_3_).

In spite of these similarities between the experiments, there are also some remarkable differences. All parameters measuring the effects of peers on first- and second-order beliefs (*β*_2_, *β*_3_) are higher in CPR-China while those of messaging (*γ*_2_ and *γ*_3_) are smaller. Without messaging, the effect of social projection (*α*_2_) is much smaller in CPR-China than in CPR-Spain. Although not statistically significant, the weights of personal norms (*B*_1_) are smaller while those of normative and empirical expectations (*B*_2_, *B*_3_) are larger in CPR-China than in CPR-Spain. With messaging, the weights of normative expectations on decision-making (*B*_2_) are larger in CPR-China. The weight of material factors *B*_0_ is smaller in CPR-China (significantly smaller in the case of no messaging). All this suggests that the higher extraction efforts *x* in CPR-China are explained not by the greater importance of material factors but by stronger conformity and reliance on observations when forming second-order beliefs. That is, larger values of *β*_2_ in CPR-China result in larger normative expectations 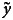, which together with larger values of weight *B*_2_, lead to larger extraction efforts *x*. The facts that both *x* and *β*_1_ are larger in CPR-China without messaging also explains why the personal norms *y* are also larger there.

Using the *k*-means method (MacQueen, 1967), we performed a cluster analysis based on the estimated coefficients of decision-making and beliefs dynamics. The method identifies a small number of interpretable clusters which are largely similar between the experiments (see Section S3.1 of the SM).

### Social value orientation

Social value orientation tests (Murphy et al., 2011; Murphy &Ackermann, 2014) allow us to separate the subjects into two types: prosocial and individualists (Table S2 in the SM). Without messaging, the differences in parameters between these two types are small in both experiments. With messaging, in CPR-Spain prosocial types have larger *B*_1_ (larger importance of personal norms) and larger values of parameters *γ*_2_, *γ*_3_ measuring the weight of messaging in the formation of second-order beliefs. In CPR-China, prosocial types have larger *B*_3_ (stronger conformity with observed peer behaviour).

Interestingly, both prosocial and individualist types are present in all behavioural clusters identified by the *k*-means method. A closer look at the differences between subject behaviour in CPR-Spain and CPR-China shows the existence of three different ‘pathways’ to being identified as a prosocial type in the SVO tests (see Section S3.5.2 of the SM). Among individuals where personal norms significantly outweigh other factors in decision-making (*B*_1_ ≈ 1), prosocial individuals exhibit greater sensitivity of personal norms to messaging (*γ*_1_ is larger). In cases where no single factor dominates decision-making, prosocial individuals in CPR-Spain tend to assign higher importance to personal norms (*B*_1_ is relatively larger), while in CPR-China they prioritise empirical expectations (*B*_3_ is relatively larger) in their decision-making process. For further information, see Section S3.5.2 of the SM.

### Rule-following

We define ‘rule-followers’ and ‘rule-breakers’ as subjects with rule compliance rates higher than 0.75 and smaller than 0.25, respectively (see SM). The frequencies of rule-followers and rule-breakers are similar in CPR-Spain and CPR-China (see Sections S.3.2 and S.3.4 for details). Interestingly, while in CPR-Spain rule-following is strongly associated with prosociality (with odds ratio of 5.0), such association is absent in CPR-China (see Section S3.5.1 of the SM).

Without messaging, the differences between rule-followers and rule-breakers in the dynamics of mean values of *x*, *y*, 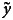 and 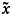 are small (see Figure S18 in the SM). With messaging, the difference in extraction efforts *x* between the two types becomes large (see Figure 4). However relative to the case of no messaging, rule-breakers greatly increase their efforts in CPR-Spain (i.e. there is a backfiring effect) while in CPR-China their efforts are not affected much by messaging. In CPR-Spain, rule-followers have somewhat lower values of *y*, 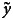 and 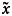 while in CPR-China the difference is noticeable only in normative expectations 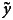.

**Figure 3.**
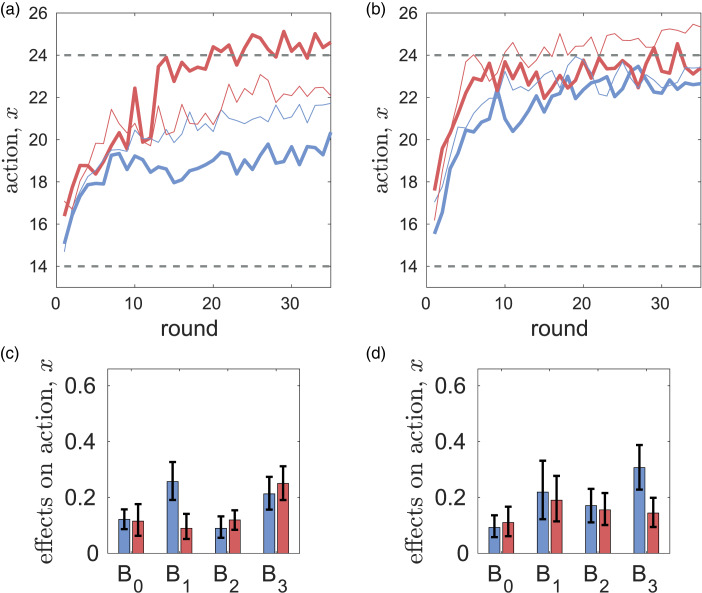
Differences in the dynamics of extraction efforts *x* (a, c) and parameters of the best response function *B*_0_, *B*_1_, *B*_2_, *B*_3_ (b, d) for individualist (bold red curves) and prosocial (bold blue curves) subjects in the CPR experiments with messaging. Thin curves show the corresponding mean extraction efforts of individualist and prosocial subjects in the case of no messaging. Dashed horizontal lines show the social optimal effort *x*_opt_ = 14, and the Nash equilibrium *x*_ne_ = 24. Parts (a) and (c) are reproduced from Figure 5 in Tverskoi et al. (2023).

**Figure 4.**
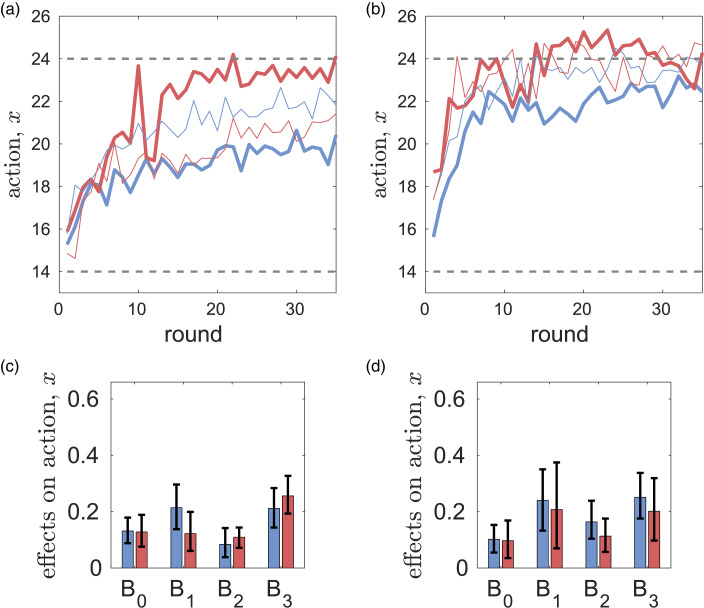
Differences in the dynamics of actions *x* (a, c) and parameters *B*_0_, *B*_1_, *B*_2_, *B*_3_ of the utility function (b, d) for rule-breakers (bold red curves) and rule-followers (bold blue curves) in the CPR experiments with messaging. Thin curves show the corresponding mean extraction efforts of individualist and prosocial subjects in the case of no messaging. Dashed horizontal lines show the social optimal effort *x*_opt_ = 14, and the Nash equilibrium *x*_ne_ = 24. Parts (a) and (c) are reproduced from Figure 6 in Tverskoi et al. (2023).

In both experiments, rule-followers have higher *B*_1_, *γ*_1_, *γ*_2_ (i.e. higher importance of personal norms for action and of messaging for first an second order beliefs). Although this observation is in line with intuition, the differences are not statistically significant. Coefficients *B*_2_, *B*_3_ measuring the importance of normative and empirical expectations are larger in rule-followers in CPR-China, but the differences are not statistically significant either. There is an association between rule-following and clusters identified by the *k*-means method (see Table S5 in the SM).

### Stubborn individuals and conditional compliers

Under conditions used in the CPR experiments without messaging, theory predicts asymptotic convergence of all the main dynamic variables (i.e. *x*, *y*, 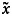, 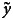) to the same value, which represents the symmetric Nash equilibrium of the model (Gavrilets, 2021). Such a convergence is clearly not happening for personal norms *y* (see Figure 1). While the above prediction assumed that all coefficients controlling beliefs dynamics were positive, so that all individuals exhibited flexibility in their beliefs, a number of subjects in our experiments did not change their personal norms. As a consequence, the average personal norm *y* will stabilise below the Nash equilibrium. In Tverskoi et al. (2023) we called such subjects ‘stubborn’. Importantly, such individuals can still change their behaviour as well as their first- and second-order beliefs.

In Section S3.6 of the SM, we explore the differences between stubborn individuals and others in more detail. The results show an interesting cultural difference. In CPR-Spain, stubborn individuals make smaller efforts *x* compared with other participants (reducing the average effort compared with the Nash equilibrium), while in CPR-China their average effort is similar to that of other participants. The reason is that in CPR-Spain personal norms play a key role in decision-making and belief formation of stubborn individuals. In contrast, in CPR-China, stubborn individuals in their decision-making largely ignore their personal norms, but put most weight on normative and empirical expectations. Messaging increases both the number of stubborn individuals and the overall stability of personal norms.

In our earlier work (Tverskoi et al., 2023), we also identified individuals whose behaviour did not fit the model well. Their dynamics of personal norm *y* and actions *x* show a steplike pattern with *x* taking relatively small values initially but then rapidly increasing to large values. It appeared that such individuals complied with messaging initially but then switched to much higher efforts after realising that others do not comply with messaging. We called such individuals ‘conditional compliers’ by analogy with ‘conditional cooperators’ who switch to defections when others defect (as observed in many behavioural experiments; Fischbacher and Gächter, 2010; Andreozzi et al., 2020).

Our results show that in their decision-making, conditional compliers put more weight on behaviour of others (parameters *B*_2_, *B*_3_), less weight on material factors (parameter *B*_0_), and typically, are characterised by lower effects of messaging (parameters *γ_i_*) on the dynamics of second-order beliefs 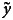 and 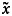) compared with the rest of the subjects. Conditional compliers are also ‘responsible’ for a step-like increase in the average value of personal norms *y* in the middle of the CPR-Spain experiment with messaging. In Section S3.7 of the SM, we explore the differences between conditional compliers and the rest of the subjects in more detail.

## Collective risk experiments

We also analysed data from two experiments which used the CR game (Milinski et al., 2008; Szekely et al., 2021; Vriens et al., 2024), a coordination game with cooperative and noncooperative Nash equilibria, frequently used to study societal threats, such as climate change, pandemics and natural disasters, which require coordinated efforts from multiple parties. The experiments were run in Spain in 2018 (Szekely et al., 2021) and 2020 (Vriens et al., 2024), and will be referred to as CR-2018 and CR-2020, respectively. Note that the two papers on the CR experiments (Szekely et al., 2021; Vriens et al., 2024) did not use our novel theoretical framework but rather focused on the effects of risk change on the strength of cooperative social norms. Contrasting the results of the CPR experiments and the CR experiments we can further test the generality of our findings. In the CR experiments, participants received a 100 point endowment each round over 28 days (one round a day). Each six-member group could prevent a disaster by collectively spending at least 300 points. If the threshold was met, the collective risk was averted and participants retained their remaining points; if not, they lost that round's earnings with probability *p*. A fair contribution of each subject to avert disaster was thus *x* = 50 points. At each round, personal normative beliefs, empirical expectations and normative expectations were elicited randomly either before or after they made their contribution decision. Questions about empirical and normative expectations were incentivised by paying the subjects based on the accuracy of their answers.

Two treatments were conducted: high–low (HL) and low–high (LH). In the HL treatment, the risk of total loss was initially set at *p* = 0.9 for the first 2 weeks and then decreased to *p* = 0.6 for the last 2 weeks, while in the LH treatment, this order was reversed. With these risk levels, contributing 50 points each round (thus averting disaster) resulted in higher expected payoffs than contributing nothing. The initial rationale behind selecting these treatments was to investigate whether stronger norms, which were anticipated to develop under higher threat levels, would render behaviour more resistant to change compared with weaker norms, expected under lower threat levels.

### Trajectories

**Figure 5.**
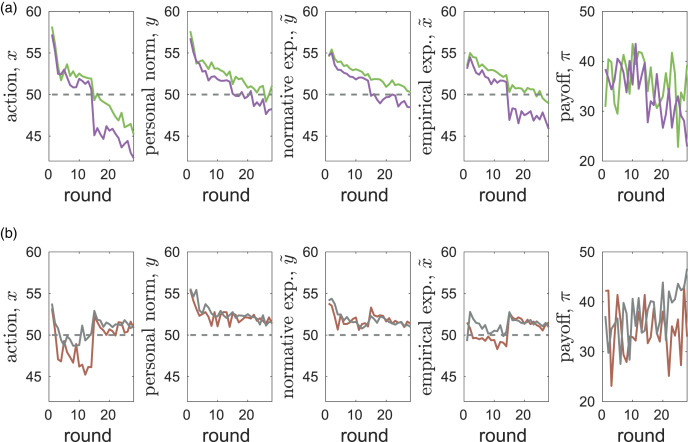
The dynamics of means of contributions *x*, personal norm *y*, normative expectation 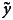, empirical expectation 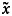, and actual material payoff *π* in the Collective Risk experiments. (a) High–low risk treatment in the two experiments: CR-2018 (green) and CR-2020 (purple). (b) Low–high risk treatment in the two experiments: CR-2018 (brown) and CR-2020 (black). The switch from one risk level to another happens before round 15. Dashed horizontal lines show the fair individual contribution *x* = 50.

We show the corresponding dynamics of our main variables in Figure 5 where we group the trajectories by the treatment. In the HL treatment, all variables continuously decline with significant drops after switching from high to low risks. These drops are much more pronounced in the CR-2020 experiment. In the LH treatment, the decline is interrupted by sudden increases in contributions *x* and second-order beliefs 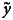 and 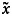 after switching from low to high risks. Changing contributions after a change in risk are intuitive. The increases in contribution *x* and empirical expectations 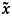 were much more pronounced in the CR-2018 experiment. Correspondingly, there are large differences between the two experiments in individual contributions during the low-risk periods: subjects in the CR-2020 experiment contribute less in the HL treatment but more in the LH treatment. A detailed comparison (Vriens et al., 2024) between the two experiments did not find a specific variable responsible for the differences between them, other than the suggestion that in 2020 the experiment was run under COVID-19 lockdown when individuals were experiencing a sudden collective threat in their daily life.

The standard deviations of the main variables follow a similar pattern to that in the CPR experiments (see Figure S3 in the SM). Interestingly, the standard deviations increase over time in the HL treatments when the risk is low. This is because some individuals continue making relatively large contributions even after a reduction in risk, while others significantly reduce their contributions (see the results of a cluster analysis in Section S3.1 of the SM).

### Parameter estimates

**Figure 6.**
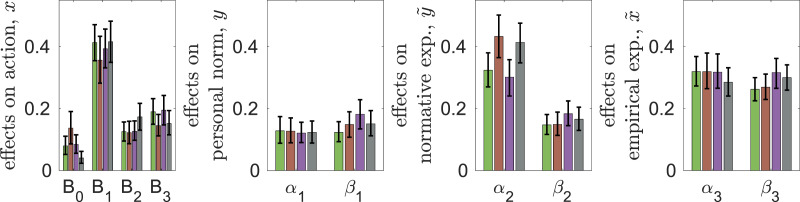
Estimates of parameters *B*_0_, *B*_1_, *B*_2_, *B*_3_ of decision-making and beliefs dynamics *α_i_*, *β_i_* (with 95% bootstrap confidence intervals) in the four Collective Risk experiments: CR-2018-HL (green), CR-2018-LH (brown), CR-2020-HL (purple) and CR-2020-LH (black).

Figure 6 shows parameter estimates. First, all parameters are statistically different from zero. Second, there is strong similarity in parameter estimates between experiments in spite of the differences between them in treatments and subject pools. Specifically, personal norms (*B*_1_) are the most important factor in decision-making while material payoffs (*B*_0_) are the least important factor (except for the CR2018-LH case where *B*_0_ is similar to *B*_2_ and *B*_3_). The effects of cognitive forces (*α*_1_, *α*_3_) and social influence (*β*_1_, *β*_3_) on personal norms *y* and empirical expectations 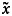 are comparable in magnitude. In contrast, the effect of social projection (*α*_2_) on normative expectations 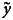 is much stronger than that of social influence *β*_2_. Under the HL treatment, the effect of conformity *B*_3_ in behaviour tends to be stronger, while that of social projection *α*_2_ in the formation of normative expectations is smaller than under the LH treatment. This may be explained by the uncertainty arising when transitioning from high to low risk levels when individuals are not certain what to do and what others will do, and therefore they rely more on the observed behaviour of others (Morris et al., 2015; Li et al., 2021).

A cluster analysis based on the estimated coefficients of the best response function and beliefs dynamics identifies a small number of clusters which are largely similar to those in the CPR experiments (see Section S3.1 of the SM).

### Social value orientation

**Figure 7.**
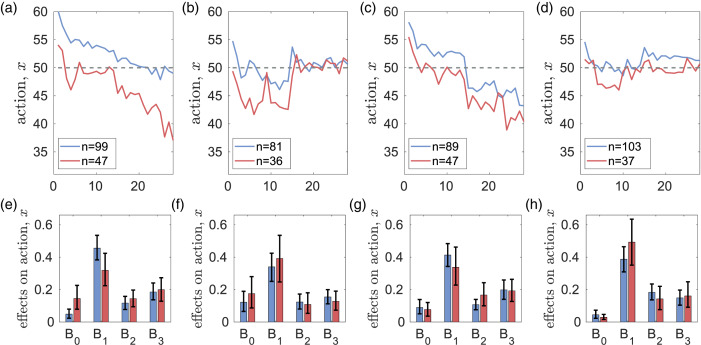
Differences in the dynamics of contributions *x* (a–d) and parameters *B*_0_, *B*_1_, *B*_2_, *B*_3_ of decision-making (e–h) for individualist (red) and prosocial (blue) subjects in the CR experiments. Dashed horizontal lines show the fair individual contribution *x* = 50.

Figures 7 and S16 in the SM show that prosocial individuals make larger contributions especially when the risk is low. However the differences in the parameters of the two types are not significant. The only exception is the CR-2018-HL experiment where prosocial individuals have significantly smaller weight *B*_0_ of material payoffs and larger weight *B*_1_ of personal norms. Associations between prosocial tendencies and behavioural clusters are shown in Table S6 in the SM.

### Rule-following

**Figure 8.**
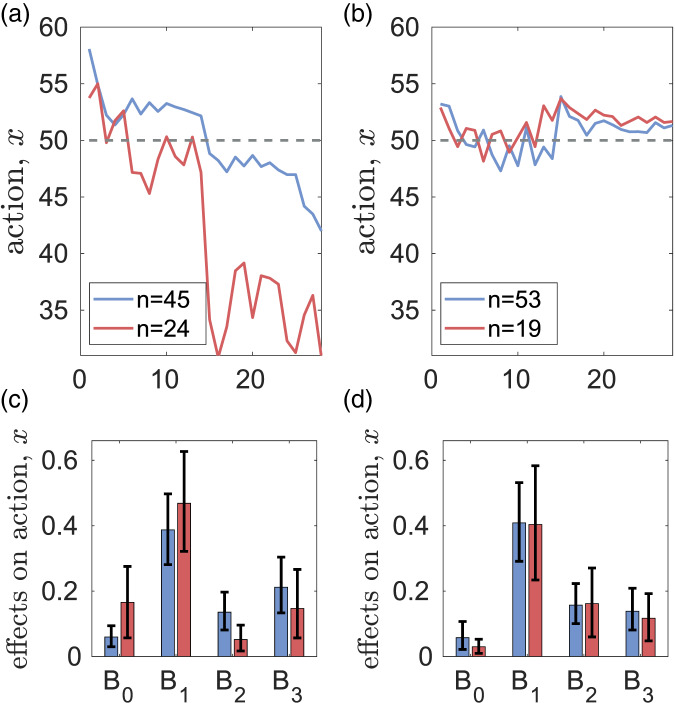
Differences in the dynamics of contributions *x* (a, b) and parameters *B*_0_, *B*_1_, *B*_2_, *B*_3_ of decision-making (c, d) for rule-breakers (red) and rule-followers (blue) in the experiments with no messaging. Dashed horizontal lines show the fair individual contribution *x* = 50.

Rule-following tests were done only in the CR-2020 experiment. Figures 8 and S18 show that in the HL treatment, rule-followers contribute more, have smaller values of *B*_0_ (material payoff) and larger *B*_2_ (normative expectations). They also have higher weight of cognitive dissonance *α*_1_ in personal norm formation. In the LH treatment, the contributions of rule-followers and rulebreakers are similar, and there are no differences in the decision-making parameters. However rule-breakers have lower weights *β_i_* of observations in belief formation (see Figure S18 in the SM).

In the case of the HL treatment, an analysis of the relationships between rule-following and clusters based on the decision-making parameters shows different ‘pathways’ for rule-following (Figure S20 in the SM). In subjects whose actions are mostly defined by personal norms (i.e, those with large *B*_1_), rule-breaking is associated with lower effects of cognitive dissonance in personal norms formation. In subjects whose actions are affected by all factors, rule-breaking is associated with stronger effects of material factors and lower effects of conformity with others (see Section S3.5.3 of the SM).

### Stubborn individuals

In the CR experiments, stubborn individuals are those who believe that the right thing to do is to make a fair contribution of *x* = 50 (i.e. their personal norms *y* are close to 50) and incorporate this belief in their decision-making and the formation of normative expectations 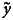. They are not willing to compensate for inadequate contributions of others and, as a result, they typically make smaller contributions *x* than most other subjects. The normative expectations of stubborn subjects are also closer to personal norms compared with other participants. For more details, see sec. S3.6 of the SM.

## Comparison of the CPR and CR experiments

**Figure 9.**
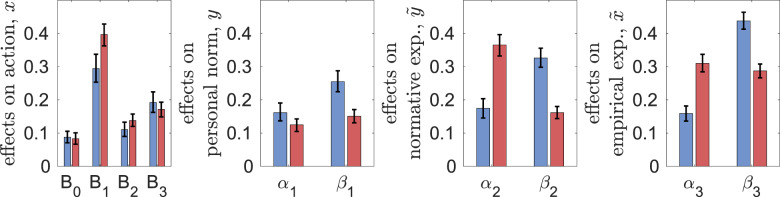
Mean parameter estimates over two CPR experiments with no messaging (blue) and four CR experiments (red) with 95% bootstrap confidence intervals.

Figure 9 illustrates the differences between the two sets of experiments in the strength of forces shaping behaviour and beliefs. In the CR experiments, the weights of personal norms and normative expectations in decision-making are significantly larger than in the CPR experiments. In belief dynamics, the influence of cognitive forces in forming normative and empirical expectations is larger, while the impact of observations on personal norms and normative and empirical expectations is smaller. Consequently, normative and empirical expectations are much closer to personal norms in the CR experiments than in the CPR experiments. Furthermore, in the CR experiments, personal norms *y* exhibit greater stability over time, as reflected in the reduced values of parameters *α*_1_ and *β*_1_. These differences are probably explained by two factors. First, in the CR experiments, it is much clearer to subjects what the ‘right thing’ to do is – contributing a fair share, or 50 units – so their personal norms are better defined than in the CPR experiments without messaging. As a result, personal norms have stronger effects on individual decision making and second-order beliefs dynamics. The second factor is that the potential consequences of antisocial behaviour are much more severe in the CR games (as subjects can lose everything) than in the CPR games. As a result, the norm of contributing a fair share is highly salient. These differences also explain the observation that while the average payoffs continuously decline in the CPR experiments, in the CR experiments this happens only during the low-risk period under the HL treatment.

## Discussion

### Introduction

We used four experiments with two treatments each, based on two social dilemmas with participants from two distinct cultural backgrounds to rigorously evaluate and compare the effects of material, social, cognitive and cultural factors in the dynamics of human behaviour and beliefs. Our integrative approach has allowed us to measure the weights of these factors on exactly the same scale and in the same experimental setup. This standardisation significantly streamlines the comparison process, ensuring that we are making like-for-like assessments. It has also allowed us to uncover some interactions between these forces. Our data strongly support this theoretical framework, demonstrating its usefulness, flexibility, generality and predictive ability, thus filling an important gap in our understanding of human behaviour. Concurrently, our model-based analysis has generated several new and valuable insights.

Across all our experiments, our findings indicate that in decision-making, personal norms held the greatest or second greatest weight, while material payoffs had the least or second least. Our results thus reinforce earlier conclusions about the significance of personal norms in shaping social behaviour (Schwartz, 1977; Gächter, 2014; Capraro & Rand, 2018; Basić & Verrina, 2020; Catola et al., 2021). Both normative and empirical expectations had intermediate weights, strengthening the argument for including beliefs, expectations and norms into game theory models Galesic et al. (2021) and Molnar and Loewenstein (2022). In the dynamics of personal norms and normative expectations, cognitive forces, such as cognitive dissonance and social projection, were found to have similar or larger effects than observations and social influence. Our results thus support the need for the integration of cognitive processes into models of human behaviour and belief dynamics (Galesic et al.^2021^, Gavrilets et al. 2024).

#### Between-individual variation

Our results echo prior studies on individual variability in behavioural responses to social beliefs (Fischbacher et al., 2001; Fischbacher and Gächter, 2010; Fehr and Schurtenberger, 2018), also showcasing individual differences in decision-making's four core factors: material aspects, personal norms, normative and empirical expectations. Additionally, we detail significant variation in belief updates and responses to messaging. We identify the presence and substantial influence of ‘stubborn’ individuals and ‘conditional compliers’ on group behaviour. Our findings further reveal considerable variation within types recognised by social value orientation tests (Murphy and Ackermann, 2014) and rule compliance tests (Kimbrough and Vostroknutov, 2018), shedding light on different pathways for classifications, such as ‘prosocial’ or ‘individualist’, and rule-follower/breaker. Notably, in some ‘prosocial’ individuals decision-making is primarily driven by personal norms, which are particularly sensitive to messaging. For other ‘prosocial’ individuals, no single factor dominates their decision-making process. However, among these individuals, personal norms carry a relatively large weight for Spanish subjects, while empirical expectations hold relatively greater importance for Chinese subjects. In the CR experiments, we found that rule-following tendencies in risky settings are expressed in situations where a period of high risk is followed by a decrease in risk. Overall, the results suggest that the evolution of rule-following and prosociality is linked to the history of environmental shocks and the evolution of cultural tightness-looseness.

#### Messaging

In the CPR experiments, messaging had a significant impact on individual actions, personal norms and both normative and empirical expectations, with the effects varying among individuals. For some, particularly individualists and rule-breakers, messaging could backfire, prompting opposite behavioural changes. This suggests that messaging effects can be overlooked if analysis solely targets average group characteristics. Without messaging, individualists and prosocial subjects, as well as rule-followers and rule-breakers, show minor behavioural and belief dynamics differences. Messaging triggers the expression of prosocial and rule-following tendencies, influencing beliefs, primarily impacting personal norms. It also appears to simplify cognitive calculations related to behavioural and belief changes, reducing the influence of cognitive factors. These findings underscore the need to consider individual heterogeneity when planning interventions and point towards the benefits of personalised strategies.

#### Effects of culture

Cultural influences significantly impact human psychology (Henrich, 2020), emphasising the importance of studying subject pools across various cultural backgrounds, including Western, educated, industrial, rich and democratic (WEIRD) and non-WEIRD groups (Henrich et al., 2010; Henrich, 2020; Muthukrishna et al., 2020). Our CPR experiments highlight key differences between Spanish and Chinese subjects, with China rated as a culturally tight country and Spain a medium one. Chinese participants generally had higher average values for all primary variables, except for personal norms when exposed to messaging. The higher extraction efforts among the Chinese are attributed more to stronger conformity and reliance on observations than material considerations. Interestingly, normative expectations had a more pronounced impact on decision-making among Chinese participants. Without messaging, social projection was less significant among Chinese than Spanish participants. As suggested earlier (Hayashi, 2021), Western individuals project onto perceived similar others, whereas Eastern individuals project onto in-group members. Our subjects, being university students from different parts of Spain and China, came with their own cultural perspectives. The difference in their responses might stem from regional cultural variations and population size in their respective countries.

Messaging and cultural backgrounds also interacted uniquely. In Spain, messaging led to reduced extraction levels among prosocial types and rule-followers, but backfired with individualist types and rule-breakers. In contrast, all Chinese participant types reduced extraction efforts, with prosocial types and rule-followers doing so more noticeably. Also, messaging was found to amplify the role of material payoffs among Chinese, but not Spanish participants. With messaging, distinctions in behaviour among rule-followers and rule-breakers, and between prosocial and individualist types, were more pronounced in Spanish than Chinese subjects. Our findings suggest that prosocial tendencies in Spanish participants manifest through adherence to personal norms, while in Chinese participants, they appear more in line with the perceived behaviour of others.

Spain and China differ not only in cultural tightness–looseness but also across a wide range of economic, ecological and cultural dimensions. Consequently, in future studies it is essential to verify the validity of our findings across subject pools from different WEIRD and non-WEIRD countries. This would help ensure that our conclusions are robust and applicable across diverse social and cultural contexts.

#### Differences with earlier experimental work

In our experiments, we directly assessed individual actions, personal norms, and normative and empirical expectations over multiple rounds as social interactions unfolded. By implementing incentivised and repeated measures of behaviour and social norms, we can accurately identify whether behavioural changes were accompanied by social norm changes. An alternative method, the strategy method, involves presenting participants with hypothetical scenarios and asking for their preferred course of action or strategy for each scenario (Fischbacher et al., 2001; Fischbacher & Gächter, 2010; Gächter et al., 2021; Gächter & Fages, 2023). By analysing participants’ responses across scenarios, researchers gain insights into decision-making processes, preferences and motivations. Although very powerful, the strategy method has limitations. Responses may be influenced by hypothetical bias, where choices differ from actual behaviour. In fact, Fischbacher and Gächter (2010) and Gächter and Fages (2023) show that predicting individual actions requires one to account for additional effects, e.g. empirical expectation, besides individual preferences elicited by the strategy method. The method assumes that participants provide truthful responses and accurately represent their beliefs. Additionally, the strategy method typically captures static preferences while preferences can evolve over time owing to observations of others’ behaviour or own past actions influencing preferences through cognitive dissonance or effort justification (Acharya et al., 2018; Andreozzi et al., 2020; Callander & Carbajal, 2022). The strategy method and multi-round experiments may yield different conclusions (Casari & Cason, 2009; Brandts & Charness, 2011; Columbus & Böhm, 2021; Burton-Chellew et al., 2022; Li et al., 2023). We opted for the latter method to directly study the joint dynamics of actions and beliefs.

Recent publications have extensively explored the impact of material payoffs and various beliefs on decision-making in social dilemmas. Normative expectations are typically assessed using the method proposed by Krupka and Weber (2013), which represents each participant's beliefs through a curve indicating the subjective appropriateness of different actions. Conversely, empirical expectations and personal norms are often quantified using a single numerical value. This disparity in measurement methods complicates direct comparisons of the relative importance of different factors, although their significance can still be inferred. Additionally, many studies employ the linear public goods game, where the optimal action maximising material payoff is zero, making it challenging to directly compare the influence of material and non-material factors. In contrast, our approach standardises all variables on the same scale, facilitating a straightforward comparison of their respective weights.

Many experiments of social dilemmas use one-shot games. Yet experimental findings indicate that subjects adjust their beliefs as social interactions unfold other multiple rounds (Ackermann & Murphy, 2019). Previous research has demonstrated that these belief updates can be effectively captured by a weighted combination of beliefs and observed peer behaviour from the preceding round (Fischbacher & Gächter, 2010). Our model extends this observation by explicitly integrating the dynamics of beliefs across multiple rounds.

The assessment of cooperative inclinations typically involves manually categorising individuals into discrete types, including conditional cooperators, free riders, triangle cooperators, unconditional cooperators and unclassified participants (Fischbacher & Gächter, 2010; Gächter & Fages, 2023). Yet social preferences may evolve over the experiment duration, as observed in cases where conditional cooperators transition into unconditional defectors (Andrews, 2020). In contrast, our methodology employs automated techniques for classifying individuals based on their responsiveness to various factors.

Despite the large number of estimated parameters, we believe that our statistically significant results are not merely due to chance for several reasons. Firstly, the consistent patterns across all four studies suggest robustness in our parameter estimates. Differences between experiments can be logically attributed to variations in the game design or cultural factors. Secondly, our analysis of prosociality and rule-following, illustrated in Figures 3 and 4, independently confirms that our parameter estimates accurately reflect the intended effects. The intuitive differences in parameter values between prosocial and individualist types, as well as between rule-followers and rule-breakers, further support this. Thirdly, our model successfully predicts the average trajectories observed in our CPR experiments, as detailed in Section S2.5 of the Supplementary Material. Observed minor discrepancies are accounted for with logical explanations (see Section S3.7 in Tverskoi et al., 2023). Finally, tests of our statistical methods on simulated data have yielded accurate estimates, reinforcing our confidence in these findings.

#### Relationship to evolutionary theories

Cooperation in social dilemmas has been a major focus of theoretical work in evolutionary biology and social sciences over the past several decades. The mechanisms and factors that have received the most attention in the literature include genetic relatedness, reciprocity, reputation, selective incentives (rewards and punishment) and cultural (group) selection (Nowak, 2006; McElreath & Boyd, 2007; Richerson et al., 2016; Schmid et al., 2021). Our experiments explicitly exclude these factors by design. Instead, cooperative tendencies in our experiments, when/if they are expressed, are driven by personal norms and social influences. However, the biological mechanisms listed above probably explain why and how personal norms and social influences (captured by model parameters *B_i_*, *β*_1_, *γ_i_*) have become important for humans (Alger & Weibull, 2013; Aoki & Feldman, 2014; Gavrilets & Richerson, 2017; Kendal et al., 2018; Akçay & Cleve, 2021; Alger et al., 2020; Alger, 2023).

We also note that although evolutionary game methods can qualitatively predict conditions for cooperation, they have some difficulties in reproducing individual behaviours, especially when subjects are heterogeneous (Wang et al., 2023). Compared with evolutionary game theory methods, our model and approach provide alternative behavioural motivations to explain empirical behavioural patterns. Our model captures individual differences in decision-making and quantitatively predicts individual behaviour in repeated interactions. This allows for a more nuanced understanding of how personal norms and social influences shape cooperative behaviour.

#### Potential applications

Our findings can enhance policy and intervention strategies by providing a theoretical and experimental foundation. Recognising individual differences in decision-making allows for tailored interventions, with financial incentives suitable for material-focused individuals and norm-based campaigns for those driven by personal norms. Identifying prosocials/individualists, rule-followers/rule-breakers, ‘stubborn’ individuals and ‘conditional compliers’ or their distributions in the populations may enable tailored interventions. Traditional mechanism design theory assumes that subjects prioritise material gains (Myerson, 1989). Our results suggest that personal norms can foster cooperative behaviour. Hence, programmes promoting personal norms and considering cultural differences can enhance communication strategies, like climate change mitigation campaigns. Our work also contributes to information design (Bergemann & Morris, 2019), emphasising the importance of considering social value orientation and rule compliance in interventions. However, the potential for messaging to backfire with individualist types underscores the need for tailored messaging. Lastly, our method examines long-term incentive effects, an area less explored compared with immediate effects (Balliet et al., 2011). Studying the temporal spillover effect of an incentive mechanism in the future could provide insights into lasting behavioural changes (Brandts & Cooper, 2006).

## Supporting information

Gavrilets et al. supplementary material Gavrilets et al. supplementary material

## Supplementary material

The supplementary material for this article can be found at https://doi.org/10.1017/ehs.2024.38.

## Research transparency and reproducibility

The data have been deposited in the Open Science Framework: CR-2018 at https://osf.io/wvgk9/; CPR-Spain at https://zenodo.org/record/7853468#.ZEL40PdOlhE; and CPR-China at https://zenodo.org/record/8050015. The data from CR-2020 will be made publicly available later. The Matlab code is available at https://zenodo.org/record/7853468#.ZEL40PdOlhE.
