# Supplementary material for: Co-evolution of behaviour and beliefs in social dilemmas: estimating material, social, cognitive and cultural determinants
Source: Evol Hum Sci. 2024 Dec 3;6:e50. doi: 10.1017/ehs.2024.38 (PMC11658954; doi:10.1017/ehs.2024.38)
Supplement: Gavrilets et al. supplementary material [file S2513843X24000380sup001.pdf]

# Co-evolution of behavior and beliefs in social dilemmas: estimating material, social, cognitive and cultural determinants

Sergey Gavrilets, Denis Tverskoi, Nianyi Wang, Xiaomin Wang, Juan Ozaita, Boyu Zhang, Angel Sánchez, and Giulia Andrighetto

*Supplementary Materials*

## Contents

**S1 Utility function in the general model** **S2**

**S2 Best response $\theta$ given empirical expectation $\tilde{x}$** **S2**

**S3 General analysis of the experiments** **S2**

S3.1 Initial values . . . S3
S3.2 Dynamics of mean values and standard deviations . . . S3
S3.3 Curve fitting: asymptotic values and the characteristic time of convergence . . . S4
S3.4 Distributions of individual estimates . . . S6
S3.5 Modeling the dynamics . . . S10

**S4 Additional results on within-population variation** **S12**

S4.1 Clustering . . . S12
S4.1.1 CPR experiments . . . S12
S4.1.2 CR experiments . . . S13
S4.2 Sizes of subgroups based on the SVO tests, rule-following tests and gender . . . S17
S4.3 Social value orientation (SVO) . . . S17
S4.4 Rule-following . . . S17
S4.5 Comparing the results of cluster analysis, SVO tests, and rule-following tests . . . S21
S4.5.1 The relationship between prosociality and rule-following . . . S21
S4.5.2 The relationship between behavioral clusters, prosocial, and rule-following tendencies in the CPR experiments with messaging . . . S21
S4.5.3 The relationship between behavioral clusters, prosocial, and rule-following tendencies in the CR experiments . . . S23
S4.5.4 The relationship between behavioral clusters, and clusters based on estimated coefficients of beliefs dynamics . . . S24
S4.6 Stubborn individuals . . . S25
S4.7 Conditional compliers . . . S27
S4.8 Individual types, cooperation, and the effect of messaging . . . S28
S4.9 Gender differences . . . S29

**S4. Instructions to subjects** **S31**

**S5. References** **S42**

# S1 Utility function in the general model

Gavrilets (2021) postulated that each individual chooses an action $x$ in an attempt to maximize the subjective utility function

$$u = \underbrace{A_0\, \pi(x,\tilde{x})}_{\text{material payoff}} - \underbrace{\frac{1}{2}\, A_1(x-y)^2}_{\text{cognitive dissonance}} - \underbrace{\frac{1}{2}\, A_2(x-\tilde{y})^2}_{\text{disapproval by peers}} - \underbrace{\frac{1}{2}\, A_3(x-\tilde{x})^2}_{\text{conformity w/ peers}} - \underbrace{\frac{1}{2}\, A_4(x-G)^2}_{\text{compliance w/ authority}}. \tag{S1}$$

That is, individuals expect to get a material payoff $\pi(x,\tilde{x})$ which depends on the expected action $\tilde{x}$ of their groupmates. They also pay psychological costs if their action $x$ deviates from what they believe is the right action ($y$) due to cognitive dissonance (Rabin, 1994), from what they think their peers and the authority expect from them ($\tilde{y}$ and $G$, respectively), and also by not conforming with the expected average behavior of their group ($\tilde{x}$). Non-negative constant parameters $A_0, \dots, A_4$ measure the weights of the corresponding terms in the utility function. The utility function (S1) was introduced as a generalization of utility functions in earlier work which included the terms accounting for material payoffs, cognitive dissonance, and conformity (Akerlof and Dickens, 1982; Calabuig *et al.*, 2018; Kuran and Sandholm, 2008; Rabin, 1994).

Assume that the partial derivative $\frac{\partial \pi(x,\tilde{x})}{\partial x}$ is a linear function of its arguments. Let $\theta$ be the action maximizing the expected material payoff $\pi(x,\tilde{x})$; in the two games used here $\theta$ can be found in a straightforward way (see below). Then the best response action can be written as a weighted sum of the values maximizing the corresponding components in the utility function and is given by equation (1) of the main text.

The advantages of estimating parameters $B_i$ of the best-response function (2) rather than parameters $A_i$ of utility function (S1) are discussed in section 4.6 of the Supplementary Material of Tverskoi *et al.* (2023a).

# S2 Best response $\theta$ given empirical expectation $\tilde{x}$

**Common Pool Resources game**. The best response action $\theta_i$ of individual $i$, given their empirical expectation $\tilde{x}_i$ about the average action of group-mates, is

$$\theta_i(\tilde{x}_i) = \begin{cases} \frac{b-c}{d} - \frac{n-1}{2}\tilde{x}_i, \text{ if } \frac{2(b-c)}{(n-1)d} \leq \tilde{x}_i \leq \frac{2}{n-1}\left[\frac{b-c}{d} - E\right], \\ 0, \text{ if } \tilde{x}_i \geq \frac{2(b-c)}{(n-1)d}, \\ E, \text{ if } \tilde{x}_i \leq \frac{2}{n-1}\left[\frac{b-c}{d} - E\right], \end{cases}$$

**Collective Risk game.** The best response action $\theta_i$ of individual $i$, given beliefs $\tilde{x}_i$ about the average action of group-mates, is

$$\theta_i(\tilde{x}_i) = \begin{cases} X_0 - (n-1)\tilde{x}_i, \text{ if } \frac{X_0 - pE}{n-1} < \tilde{x}_i < \frac{X_0}{n-1}, \\ 0, \text{ otherwise.} \end{cases}$$

# S3 General analysis of the experiments

The experimental protocols used are described in details in our earlier papers on the Common Pool Resources game (Tverskoi *et al.*, 2023b) and the Collective Risk game (Szekely *et al.*, 2021; Vriens *et al.*, 2023)

game. The estimation procedures are described in details in (Tverskoi *et al.*, 2023b). We refer the reader to these publications.

## S3.1 Initial values

Because we are dealing with transient dynamics, considering potential effect of initial conditions is important. The differences in average initial values (i.e., in round 1) of the main variables among the CPR experiments are not large (Figure S1) although in CPR-China initial values of $x$ are larger but initial values of $y$ are smaller than those in CPR-Spain. In RC experiments, the initial values of $x$ and $y$ in the HL treatments are larger than in the LH treatments as expected.

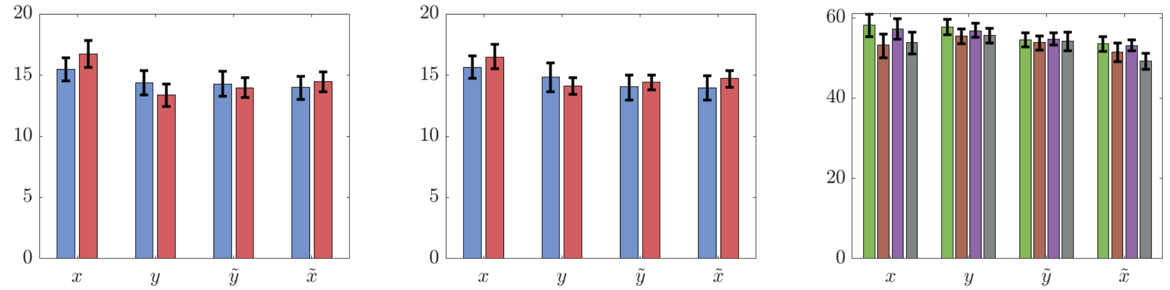

(a) CPR experiments: no messaging (b) CPR experiments: with messaging (c) CR experiments

**Figure S1:** Mean initial values of main variables: effort $x$, personal norm $y$, normative expectation $\tilde{y}$, and empirical expectation $\tilde{x}$ in: (a) the two CPR experiments with no messaging; (b) the two CPR experiments with messaging; and (c) the four CR experiments.

## S3.2 Dynamics of mean values and standard deviations

Figures S2 for the CPR experiments and S3 for the CR experiments describe the dynamics of the means and standard deviations of the main variables. Notice that variances in initial values of first- and second-order beliefs $y, \tilde{y}$ and $\tilde{x}$ are smaller in CPR-China than in Spain. However in CPR-Spain they all quickly drop after the first 2 rounds.

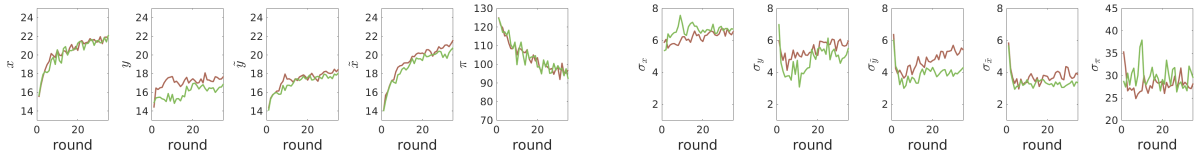

(a) CPR-Spain (b) CPR-Spain

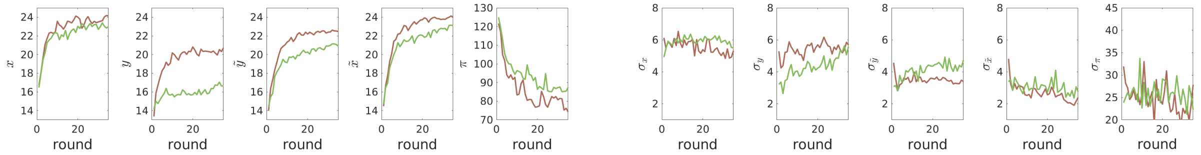

(c) CPR-China (d) CPR-China

**Figure S2:** The dynamics of means (left panels) and standard deviations (right panels) of main variables: effort $x$, personal norm $y$, normative expectation $\tilde{y}$, empirical expectation $\tilde{x}$, and actual material payoff $\pi$ in the CPR experiments: (a,b) CPR-Spain, (c,d) CPR-China. In each panel, the treatments without (brown) and with (geen) messaging are shown. Parts (a) and (b) are reproduced from Figure 1 in Tverskoi *et al.* (2023b).

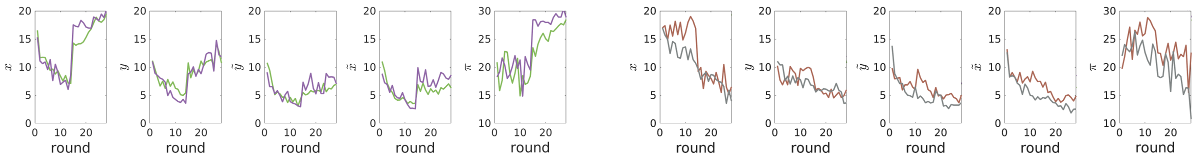


(a) HL (b) LH

**Figure S3:** The dynamics of standard deviations of investment $x$, personal norm $y$, normative expectation $\tilde{y}$, empirical expectation $\tilde{x}$, and actual material payoff $\pi$ in the Collective Risk experiments. (a) High-low risk treatment in the two experiments: CR-2018 (green) and CR-2020 (purple) (b) Low-high risk treatment in the two experiments: CR-2018 (brown) and CR-2020 (black).

## S3.3 Curve fitting: asymptotic values and the characteristic time of convergence

We can get some additional preliminary ideas about the dynamics of $x, y, \tilde{y}$ and $\tilde{x}$ by doing some simple curve fitting. We fit the following model to the data on each of our four main dynamic variables:

$$z_t = z^* + (1-v)^t(z_0 - z^*) + \varepsilon, \tag{S2}$$

where $z^*$ is a value to which the trajectory converges, $v$ is a parameter measuring the speed of convergence, and $\varepsilon$ is a random error which is assumed to have zero mean and standard deviation $\sigma$. We estimate $z^*$, $v$ and $\sigma$ for each variable separately.

The deterministic part of equation (S2) represents a solution to the linear recurrence equation

$$z_{t+1} = z_t + v(z^* - z_t).$$

which predicts an exponential convergence to an equilibrium $z^*$ at speed $v$ (assuming that the speed parameter $|v| < 1$). Rather than reporting the values of $v$, below we show the characteristic time of convergence, $\tau = ln(2)/v$.

We used nonlinear curve fitting function *lsqcurvefit* in Matlab. Table S1 summarizes the estimates of equilibrium values and the characteristic time of convergence while Figure S4 shows the fitted trajectories.

**Table S1:** Asymptotic values and characteristic time of convergence. The results with conditional compliers excluded are shown in parentheses.

| Experiment | Treatment | Asymptotic values for | | | | Characteristic time $\tau$ for | | | |
|---|---|---|---|---|---|---|---|---|---|
| | | $x$ | $y$ | $\tilde{y}$ | $\tilde{x}$ | $x$ | $y$ | $\tilde{y}$ | $\tilde{x}$ |
| CPR-Spain | no messaging | 21.6 | 17.4 | 18.0 | 21.0 | 5.4 | 2.3 | 5.0 | 6.5 |
| | w/ messaging | 21.7 | 17.2 | 17.8 | 20.4 | 6.4 | 16.2 | 5.8 | 6.6 |
| | | (20.1) | (15.7) | (17.2) | (20) | (3.1) | (1.7) | (4.5) | (5.9) |
| CPR-China | no messaging | 23.9 | 20.4 | 22.5 | 24.0 | 3.6 | 3.5 | 3.9 | 4.2 |
| | w/messaging | 22.8 | 16.3 | 20.7 | 22.5 | 3.8 | 3.3 | 4.2 | 4.2 |
| | | (22.4) | (16.2) | (20.5) | (22.5) | (3.0) | (2.9) | (3.9) | (4.0) |

The asymptotic values of all variables are larger in the CPR-China than in the CPR Spain (Table S1). In the CPR-Spain experiment, there is not much difference between the asymptotic values without and with messaging (Table S1). In the CPR-China experiment, there are significant differences between the estimates of equilibrium values in the experiments with and without messaging. Without messaging, the equilibrium values of $x = 23.8$ is very close to the Nash equilibrium at $x = 24$.

We did not attempt to fit the trajectories in the CR experiments because of shorter length of these experiments and the change in experimental conditions in the middle of each experiment.

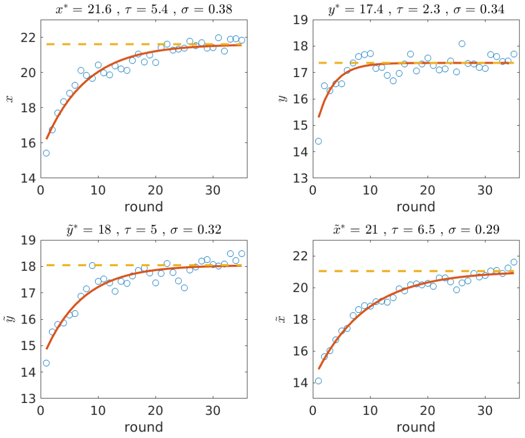


(a) Spain: no messaging

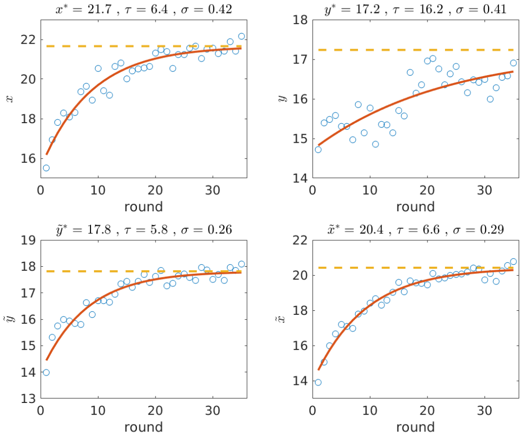


(b) Spain: with messaging

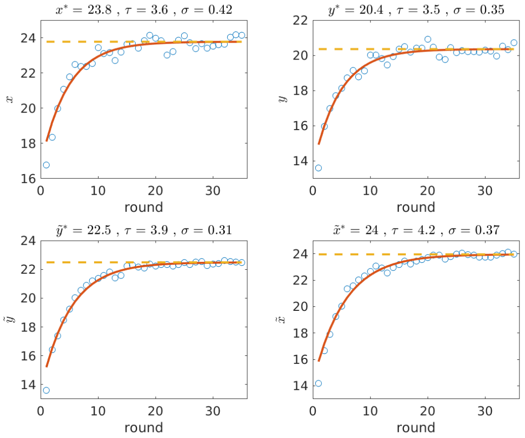


(c) China: no messaging

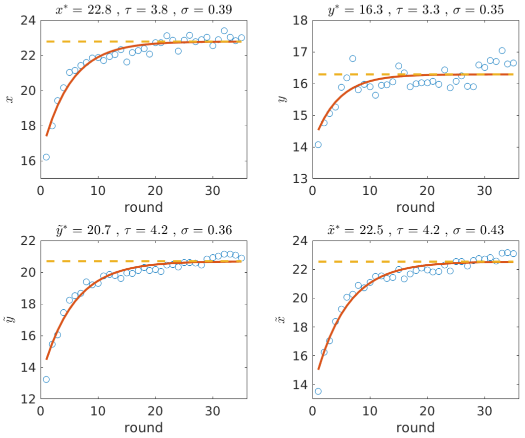


(d) China: with messaging

**Figure S4:** Fitting model (S2) to the average values of $x, y, \tilde{y}$ and $\tilde{x}$. The numbers on top are the estimated equilibrium (also shown by a dashed line), the characteristic time of convergence $\tau$, and the standard error $\sigma$. Parts (a) and (b) are reproduced from Figures S2-S3 in Tverskoi *et al.* (2023b).

Without messaging, the characteristic time of convergence is the longest for empirical expectations, while it is the shortest for personal norms. The result is intuitive: personal norms capture individual personal values which tend to be less affected by the social environment. Conversely, empirical expectations are affected by efforts of others, normative expectations, and personal norms (through normative expectations). As a result, it is natural to assume that empirical expectations equilibrate after all other variables do. With messaging, the characteristic time of convergence for personal norms is the longest in the CPR-Spain, but is the smallest in the CPR-China. This surprising effect in the CPR-Spain is associated with conditional compliers. After removing them in both the experiments, the time of personal norms convergence reduces dramatically in the CPR-Spain. Overall, without conditional compliers, the results are similar to those observed in the experiments without messaging.

## S3.4 Distributions of individual estimates

The graphs in this section show that the distributions are highly asymmetric.

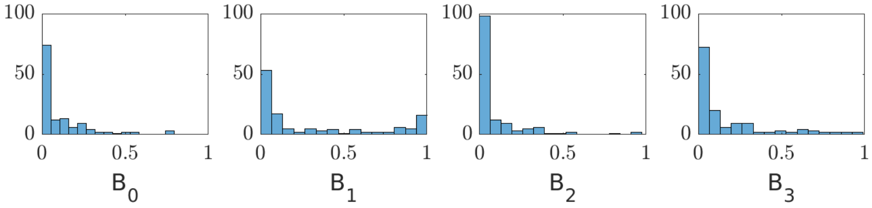


(a) Spain: no messaging

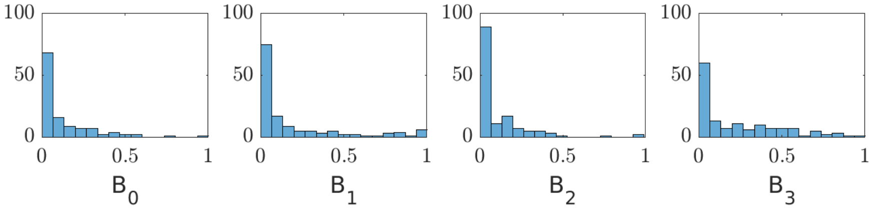


(b) Spain: messaging

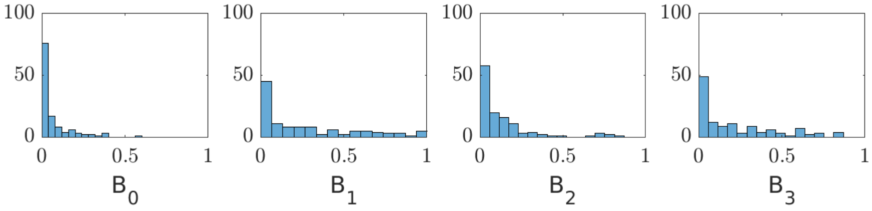


(c) China: no messaging

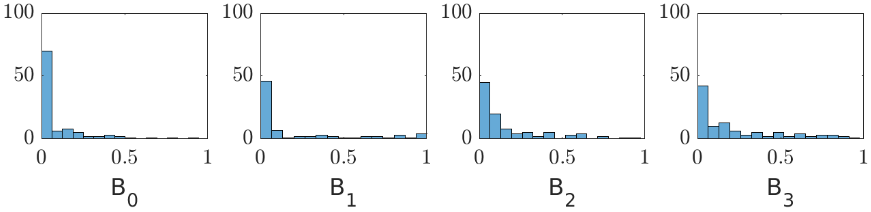


(d) China: messaging

**Figure S5:** Histograms of individual estimates of best-response parameters $B_i$ in the 4 CPR experiments. Parts (a) and (b) are reproduced from Figure S9 in Tverskoi *et al.* (2023b).

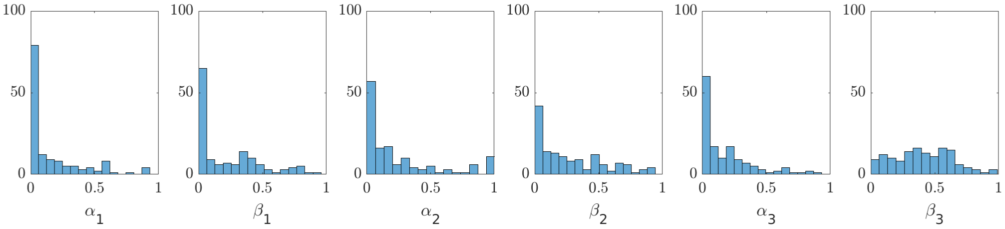


(a) Spain: no messaging

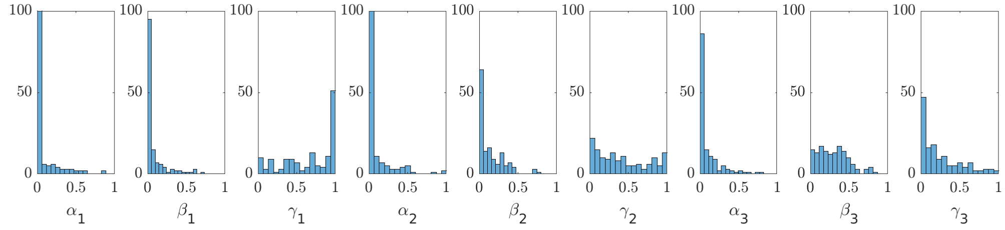


(b) Spain: messaging

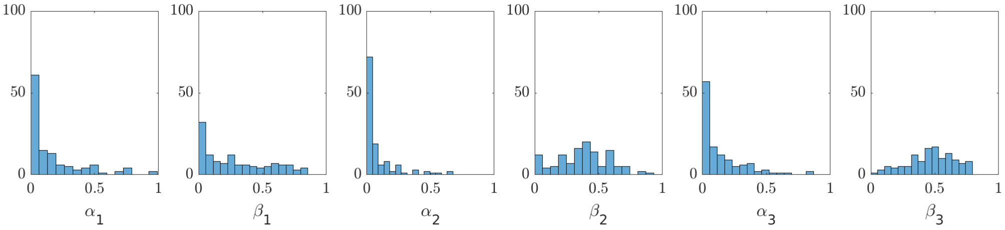


(c) China: no messaging

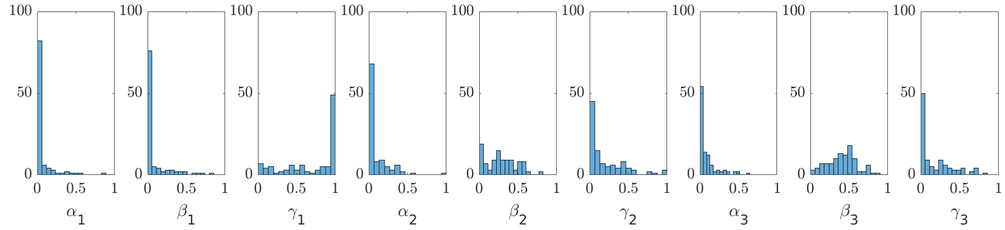


(d) China: messaging

**Figure S6:** Histograms of individual estimates of beliefs dynamics parameters $\alpha_i, \beta_i, \gamma_i$ in the 4 CPR experiments. Parts (a) and (b) are reproduced from Figure S10 in Tverskoi *et al.* (2023b).

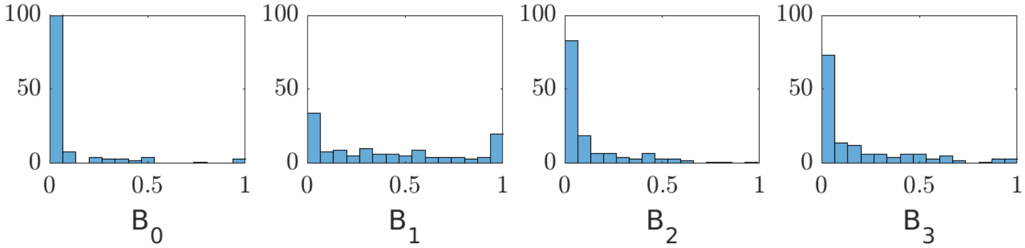


(a) CR-2018-HL

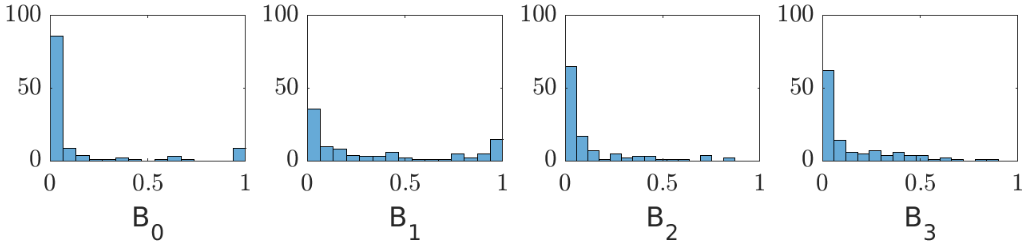


(b) CR-2018-LH

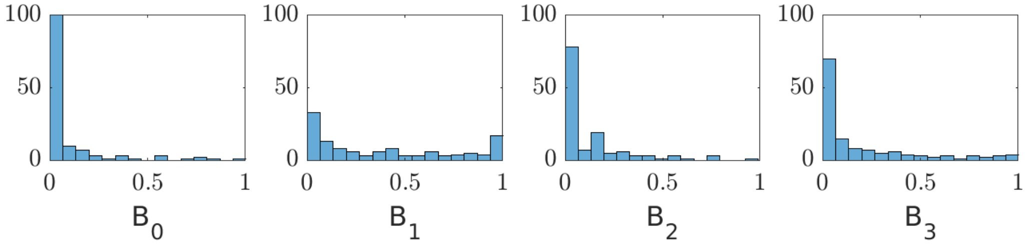


(c) CR-2020-HL

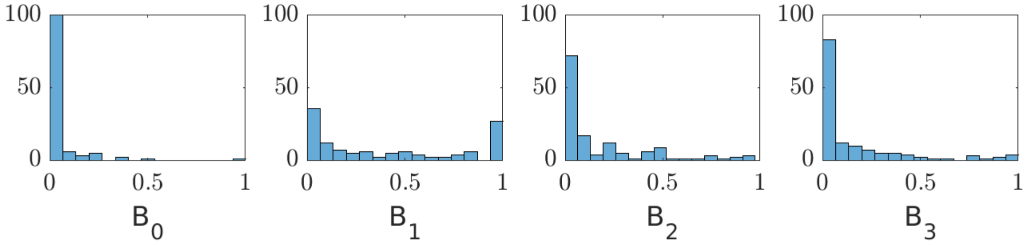


(d) CR-2020-LH

**Figure S7:** Histograms of individual estimates of best-response parameters $B_i$ in the 4 CR experiments.

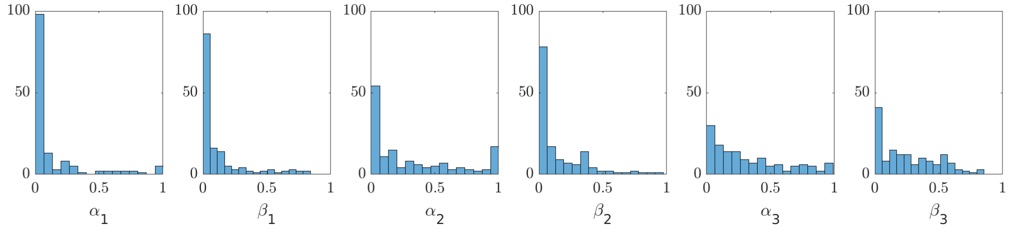


(a) CR-2018-HL

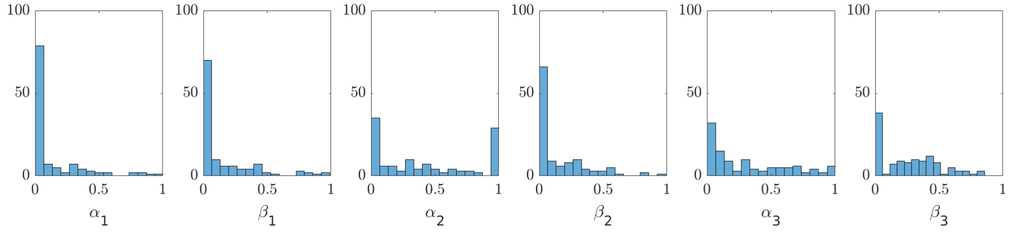


(b) CR-2018-LH

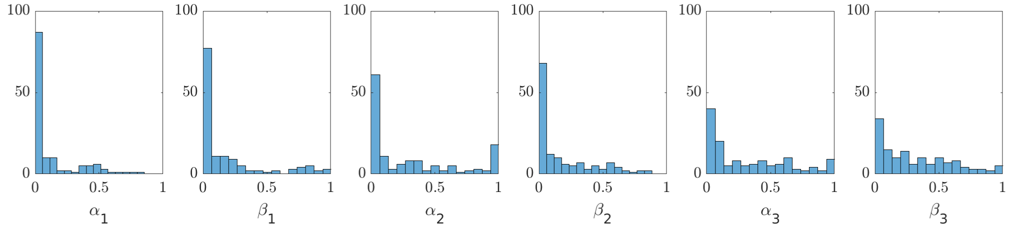


(c) CR-2020-HL

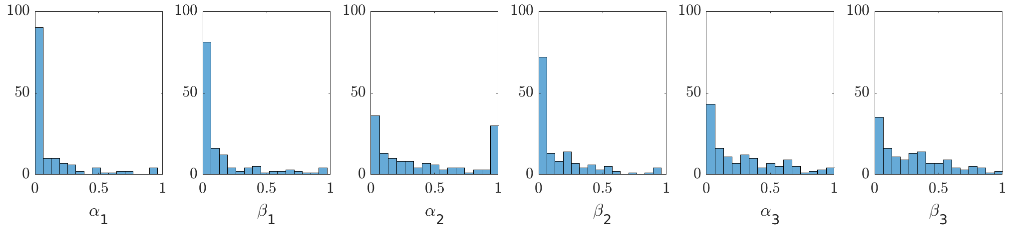


(d) CR-2020-LH

**Figure S8:** Histograms of individual estimates of beliefs dynamics parameters $\alpha_i, \beta_i$ in the 4 CR experiments.

### S3.5 Modeling the dynamics

As a further test of our approach and its predictive ability we iterated the model's dynamic equations (2-3) using estimated individual parameters. The results are illustrated in Figures S9 for the CPR-Spain (Tverskoi *et al.*, 2023b) and S10 for the CPR-China. In these figures, to obtain "predicted" trajectories we used the actual individual data in each round $(\theta, y, \tilde{y}, \tilde{x}, X)$ to predict their values in the next round. The "simulated trajectories" were obtained by repeatedly iterating the dynamic equations for 34 rounds forward using the actual individual data observed in the first round. In simulations, we reshuffled individuals between the groups randomly without attempting to recreate the exact history of individual movements between different groups; the results shown are the averages over 500 runs. Overall, given all the stochasticity and estimation errors involved, the match between the observed, predicted, and simulated trajectories is rather good. As discussed in (Tverskoi *et al.*, 2023b), the mismatch is caused by individuals for whom the dynamics of efforts are described by an S-shaped function with relatively low efforts initially and a sharp transition to relatively high efforts in the middle of the the experiment when the average contributions of others exceed a certain threshold. Since the behavior of such individuals, who we call conditional compliers, is not well described by our linear best response function (2) , this leads to the mismatch between the observed and simulated average trajectories. For more details on conditional compliers see Section S4.7 in SM of (Tverskoi *et al.*, 2023b).

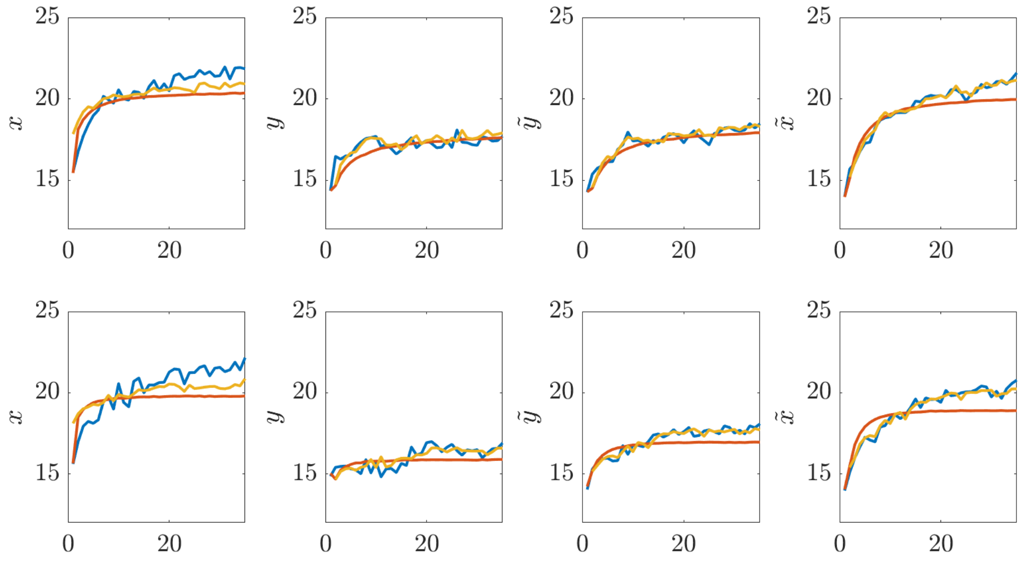


**Figure S9:** A comparison between observed (blue), simulated (red), and predicted (yellow) mean trajectories in the CPR-Spain experiments without messaging (the first row) and with messaging (the second row). The figure is reproduced from Figure 7 in Tverskoi *et al.* (2023b).

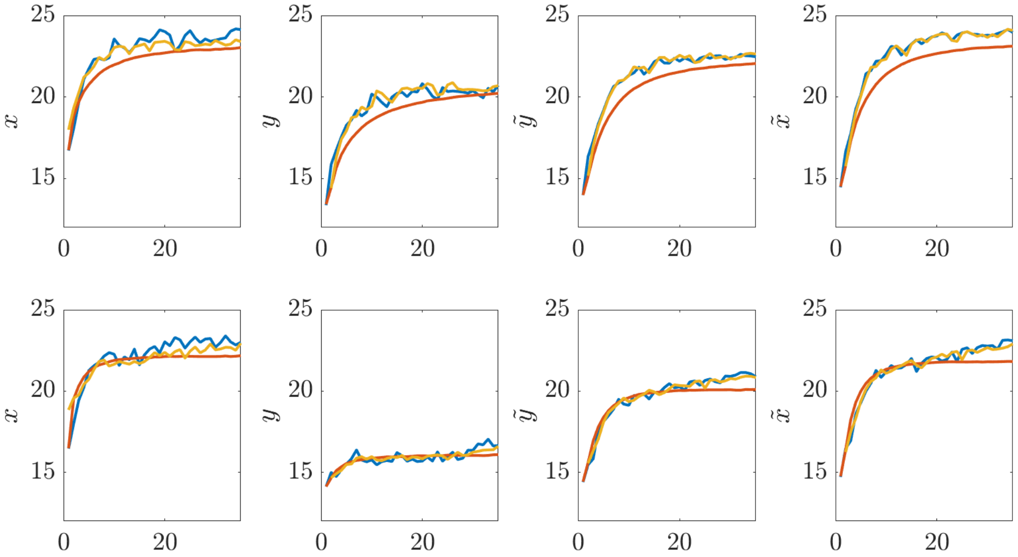


**Figure S10:** A comparison between observed (blue), simulated (red), and predicted (yellow) mean trajectories in the CPR-China experiments without messaging (the first row) and with messaging (the second row).

## S4 Additional results on within-population variation

Here we provide more detailed results on clustering, on the differences between different subgroups identified by the SVO and rule-following tests and between the genders.

### S4.1 Clustering

Using the k-means method (MacQueen, 1967), we performed a cluster analysis based on the estimated coefficients of the best response function and beliefs dynamics.

#### S4.1.1 CPR experiments

In the case of best response function parameters, the method identifies 3 clusters in CPR-Spain and 2 clusters in CPR-China. There is always a cluster of subjects with large values of $B_1$ whose decision-making is mostly driven by personal norms (Cluster 1, blue color in Figure S11); these subjects make the smallest efforts $x$ in the extraction of resources. There is little mismatch between their actions and first and second order beliefs. Moreover their $\gamma_1$ are smaller and personal norms $y$ are less affected by messaging than in individuals from other clusters. In the other two clusters there is a strong mismatch between actions and personal norms with the latter being strongly affected by messaging. For individuals in both these clusters material forces ($B_0$) are much more important than in the first cluster; and individuals make much larger efforts. One of these, which exists always and is the largest, has subjects for whom no single factor dominates others in decision-making (Cluster 2, green color in Figure S11). Typically, coefficients $B_2$ and $B_3$ in this cluster are larger than those in the first cluster. In CPR-Spain there is a third cluster of individuals with very large $B_3$ whose behavior is most strongly affected by that of their peers (Cluster 3, yellow color in Figure S11). These individuals make the largest extraction effort $x$ by the end of the experiment.

We also applied the k-means method to the coefficients of beliefs dynamics. In both experiments without messaging, three clusters differing in the forces controlling the dynamics of personal norms are observed (for details see Figure S12): those for whom cognitive dissonance is most important ($\alpha_1$ is large), those for whom observed peer behavior is most important ($\beta_i$ are large), and those for whom both cognitive forces ($\alpha_i$) and the effects of others ($\beta_i$) are comparable in magnitude. In CPR-Spain, there is an additional cluster of subjects for whom social projection is the most important force in controlling the dynamics of empirical expectations ($\alpha_2$ is large).

With messaging, there are two clusters in both experiments (see Figure S12). One cluster represents individuals whose personal norms are mostly affected by messaging (for them $\gamma_1$ is much larger than $\alpha_1$ and $\beta_1$). Their personal norms $y$ are stable and relatively low throughout the experiment. The other cluster represents subjects for whom the effects of cognitive forces, peer behavior, and messaging on personal norms are comparable. Interestingly, although the personal norms of individuals in the first cluster are close to the socially optimal value promoted by the messaging, their personal norms play a smaller role in their decision-making compared to individuals from the second cluster. This results in a small difference in average actions between the two clusters.

An interesting question is to what extent the two classifications (based on the best response parameters $B_i$ and on the belief dynamics parameters $\alpha_i, \beta_i, \gamma_i$) overlap. Given the number of clusters observed, the sample sizes of our experiments are not large enough for definite conclusions. Nevertheless our data show that in the CPR-Spain experiment without messaging, the personal norms of subjects whose decision-making is strongly affected by empirical expectations (with large $B_3$) are weakly affected by cognitive dissonance (have small $\alpha_1$). Conversely, subjects with large $\alpha_1$ have small $B_3$. In the experiments with messaging, the personal norms of subjects with large weight $B_1$ of personal norms are less affected by

messaging (their $\gamma_1$ is small). Conversely, the personal norms of subjects with low $B_1$ are strongly affected by messaging (their $\gamma_1$ is large). See section S4.5.4 for details.

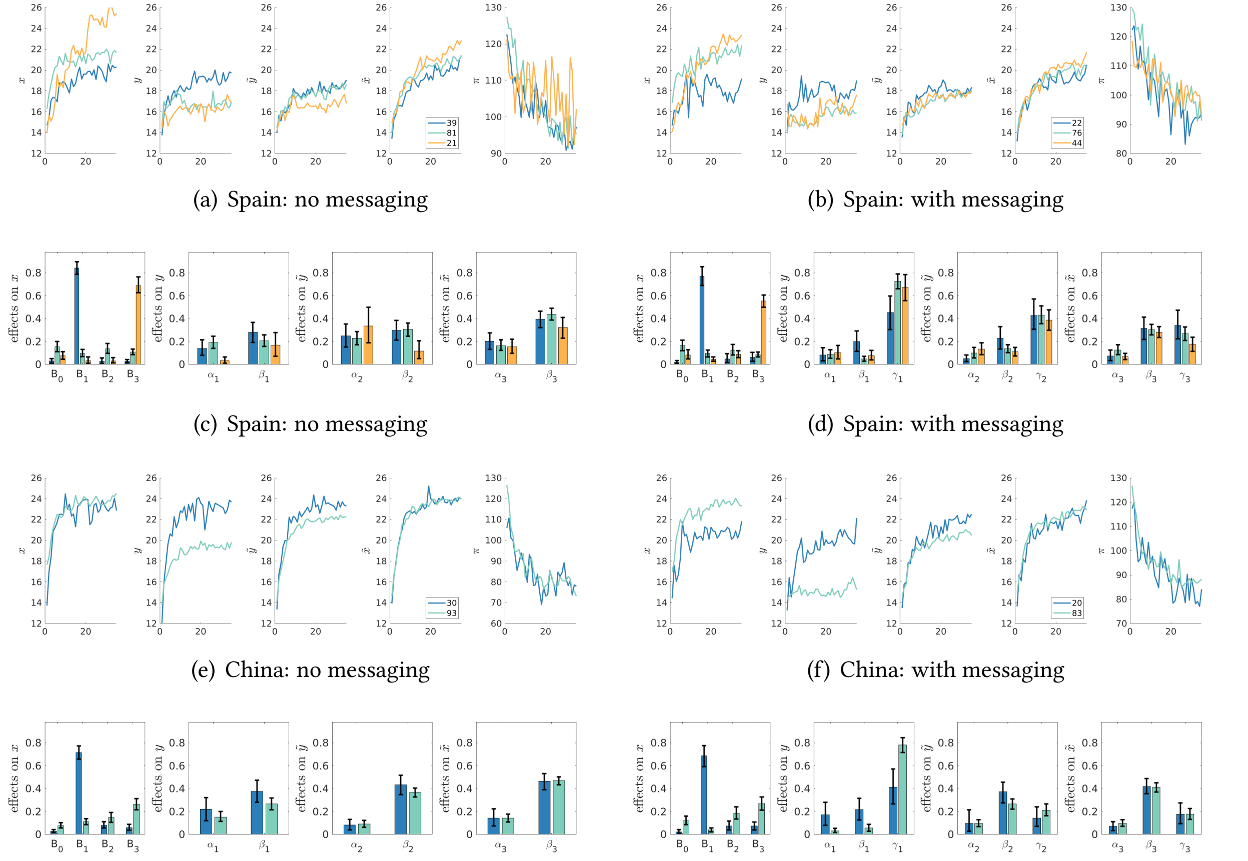

(a) Spain: no messaging

(b) Spain: with messaging

(c) Spain: no messaging

(d) Spain: with messaging

(e) China: no messaging

(f) China: with messaging

(g) China: no messaging

(h) China: with messaging

**Figure S11:** Average trajectories and parameters for individuals from k-means clusters based on estimated coefficients of utility function. Parts (a-d) are reproduced from Figure S12 in Tverskoi *et al.* (2023b).

### S4.1.2 CR experiments

As we did with the CPR experiments, we have carried out a clustering analysis of the participants in the CR experiments. The k-means method identifies 2 or 3 clusters based on the estimates of best response function parameters. Two clusters are common to all CR experiments and are similar to the two clusters common to all CPR experiments. One of them represents subjects with large values of $B_1$ whose decision-making is mostly driven by personal norms (Cluster 1, blue color in Figure S13). These individuals typically make the largest contributions. The second cluster represents subjects for whom no single factor dominates others in decision-making (Cluster 2, green color in Figure S13). There is also an additional cluster in CR-2018 under the LH treatment with individuals whose decisions are mostly driven by expected material payoffs (Cluster 3, yellow color in Figure S13). These are individuals who make very low contributions during the low-risk period but then increase them slightly above 50 in the high-risk period.

Clustering parameters of beliefs dynamics identifies 3 clusters (Figure S14). There is always a cluster of individuals with very large weight $\alpha_2$ of social projection. These subjects do not change much their personal norms $y$ over time (their $\alpha_1$ and $\beta_1$ are small) which stay slightly above 50 during the whole

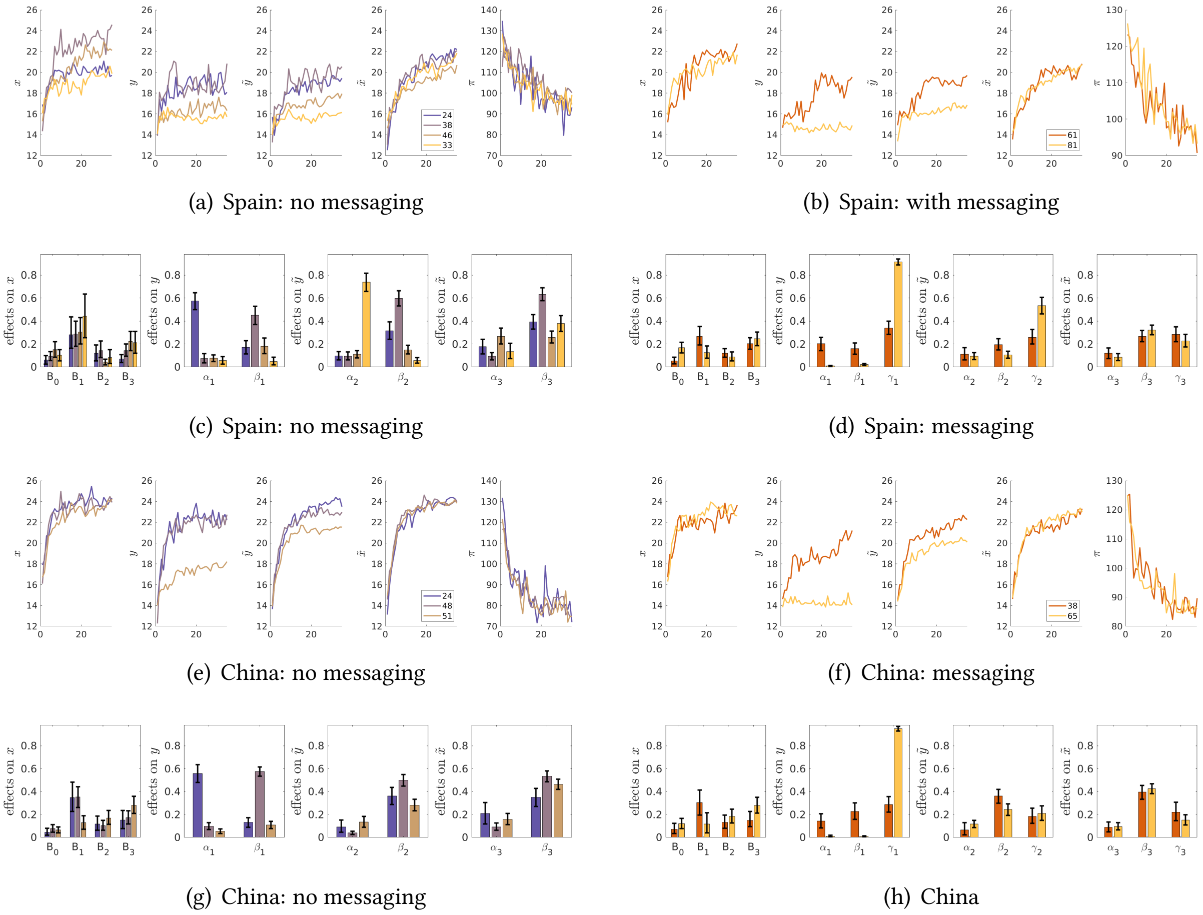


**Figure S12:** Average trajectories and parameters for individuals from k-means clusters based on estimated coefficients of beliefs dynamics. Clusters are ordered and colored according to the average value of parameter $\gamma_1$ in the experiments with messaging: clusters with higher average $\gamma_1$ are colored lighter yellow. Parts (a-d) are reproduced from Figure S15 in Tverskoi *et al.* (2023b).

experiment, and their normative expectations stay very close to personal norms. These subjects are also characterized by higher weights of cognitive factors compared to observations of others in their empirical expectation formation (i.e., $\alpha_3 > \beta_3$). Under the LH treatment, these individuals have the smallest personal norms. In CR-2018-LH, they also have the highest weight of material factors in decision making among other clusters (i.e., $B_0$ is the highest), which results in the smallest contributions $x$. Subjects of the second cluster are those whose beliefs are mostly influenced by cognitive forces ($\alpha_i > \beta_i$). Typically, they have the highest average contributions among the three clusters. The third cluster represents subjects whose beliefs are mostly influenced by observed behavior of peers ($\alpha_i < \beta_i$). The personal norms $y$ and contributions $x$ of these subjects drop most dramatically throughout the HL treatment of the experiment.

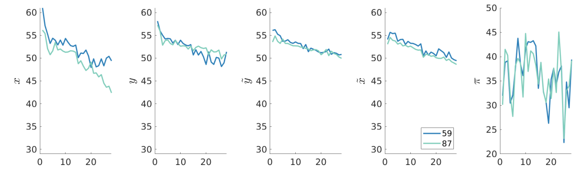

(a) CR-2018-HL

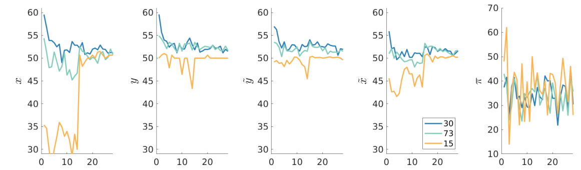

(b) CR-2018-LH

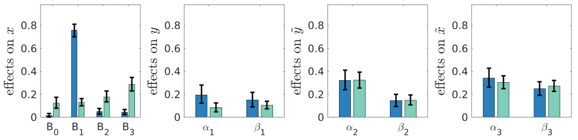

(c) CR-2018-HL

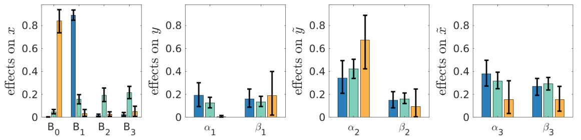

(d) CR-2018-LH

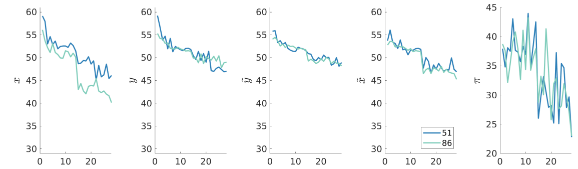

(e) CR-2020-HL

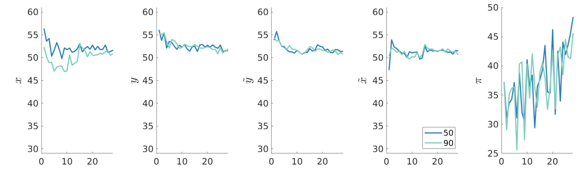

(f) CR-2020-LH

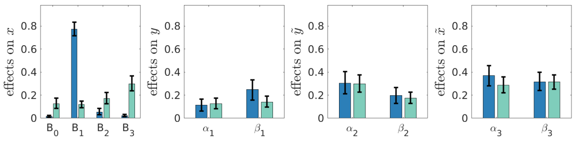

(g) CR-2020-HL

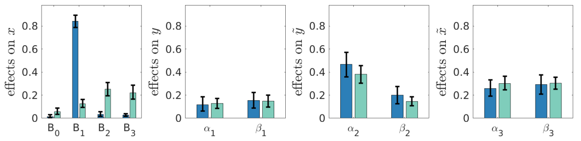

(h) CR-2020-LH

**Figure S13:** Average trajectories and parameters for individuals from k-means clusters based on estimated coefficients of utility function.

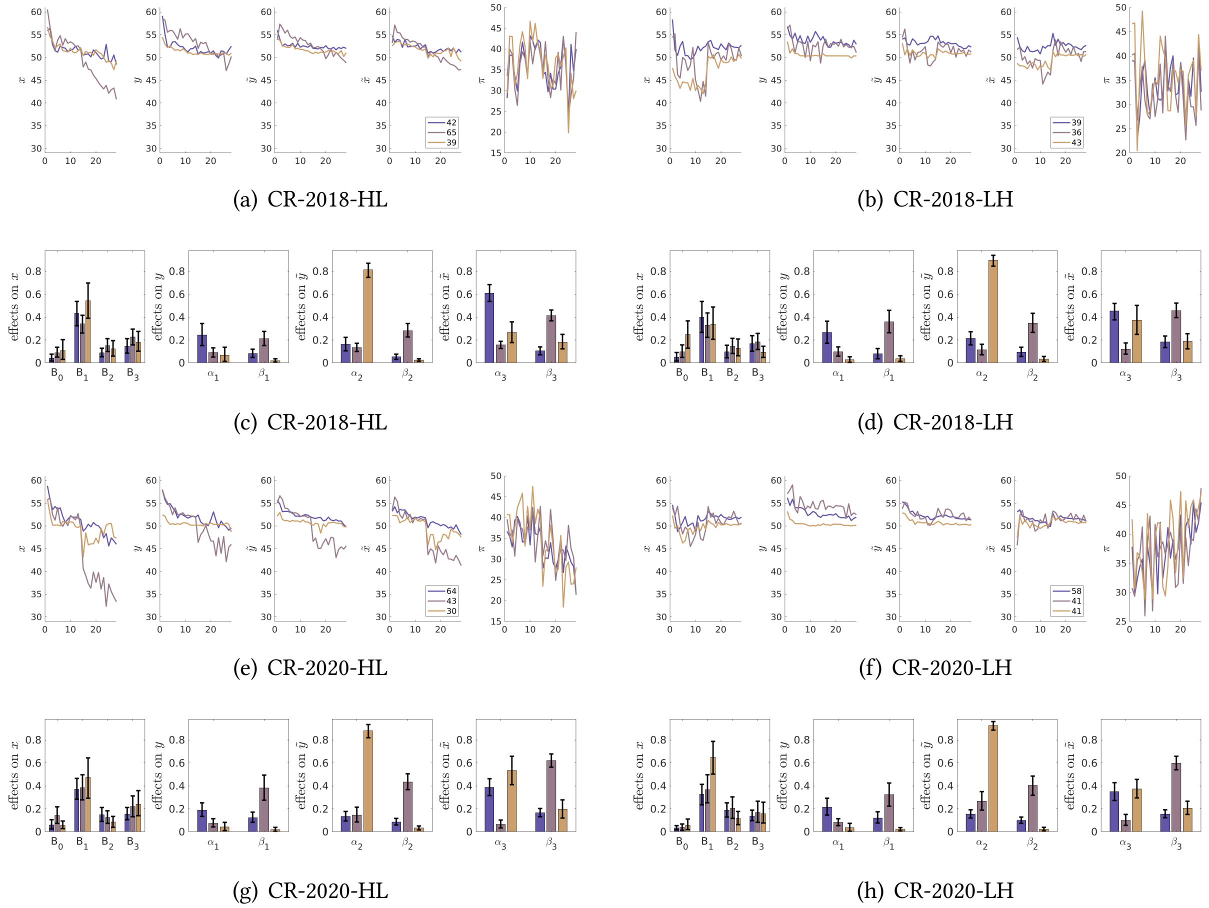

(a) CR-2018-HL (b) CR-2018-LH

(c) CR-2018-HL (d) CR-2018-LH

(e) CR-2020-HL (f) CR-2020-LH

(g) CR-2020-HL (h) CR-2020-LH

**Figure S14:** Average trajectories and parameters for individuals from k-means clusters based on estimated coefficients of beliefs dynamics.

## S4.2 Sizes of subgroups based on the SVO tests, rule-following tests and gender

| Experiment | Sample size | SVO | | Rule-following | | Gender | |
|---|---|---|---|---|---|---|---|
| | | prosocial | individualist | rule-followers | rule-breakers | males | females |
| CPR-Spain | 141 | 93 | 48 | 60 | 34 | 59 | 81 |
| | 142 | 83 | 59 | 61 | 49 | 63 | 77 |
| CPR-China | 123 | 77 | 46 | 55 | 29 | 28 | 94 |
| | 103 | 55 | 48 | 47 | 24 | 24 | 79 |
| CR-2018-HL | 146 | 99 | 47 | - | - | 60 | 86 |
| CR-2018-LH | 118 | 81 | 36 | - | - | 54 | 64 |
| CR-2021-HL | 137 | 89 | 47 | 45 | 24 | 60 | 76 |
| CR-2021-LH | 140 | 103 | 37 | 53 | 19 | 73 | 66 |

**Table S2:** Number of different categories of individuals. The rule-following tests were not conducted in the CR-2018. The table does not include the subjects who fall in the middle between "rule-followers" and "rule-breakers" in the rule-following tests, who are classified as "altruists" or "competitive" in the SVO tests, and those who did not specify their sex or identified as non-binary.

## S4.3 Social value orientation (SVO)

Subjects of both CPR experiments and those of CR-2020 experiments also completed a standard Social Value Orientation (SVO) test (Murphy and Ackermann, 2014; Murphy *et al.*, 2011) before the main experiment. In the SVO test, subjects are asked to make 6 decisions in a series of incentivized Dictator games to allocate money between themselves and a randomly assigned anonymous partner. These choices are then converted in a continuous measure of other-regarding preferences which is used to classify subjects into four different types labeled as altruists, prosocials, individualists and competitive types. With very rare exceptions (see Table S2 in SM), our subjects were classified into just two types: individualists (i.e. those who maximize the payoff to themselves) and prosocials (i.e., those who maximize the joint payoff or minimize the difference between payoffs in the Dictator game).

## S4.4 Rule-following

Participants of CPR experiments and those of CR-2020 experiments also participated in rule-following tests (applied before the main experiments). Following Kimbrough and Vostroknutov (2018), participants were tasked with dragging and dropping balls one by one into either a yellow or a blue bucket through a computer interface. For each ball placed in the yellow bucket, they received 10 cents, while each ball placed in the blue bucket earned them 5 cents. The total earnings in this task were the sum of the earnings from each bucket. The position of the buckets on the screen was randomly assigned for each individual and for each round. The instructions clearly stated that "the rule [was] to put the balls into the blue bucket". Participants were given 20 balls to allocate, so their earnings could range from 1 euro if they followed the rule completely to 2 euros if they broke the rule with each ball. The number of balls they placed in the blue bucket allowed us to quantify the degree to which every participant followed the rule. The rule compliance rate is defined as the ratio of the number of balls a participant put into the blue bucket to the total number of balls placed by the participant.

In Spanish samples (CPR-Spain and CR-2020), the distributions of the rule compliance rates appear to be similar across experiments (and similar to those reported by Kimbrough and Vostroknutov (2016). In Chinese subjects, there are only few subjects exhibiting zero rule compliance.

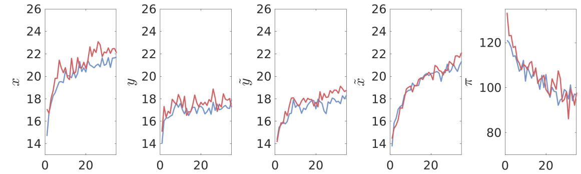

(a) Spain: no messaging

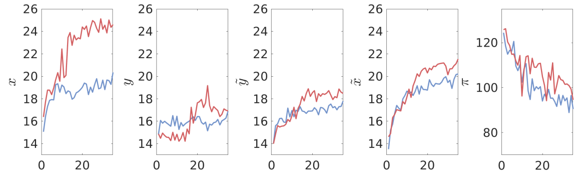

(b) Spain: with messaging

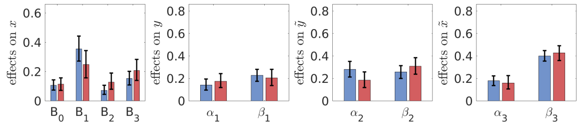

(c) Spain: no messaging

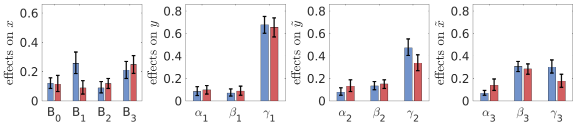

(d) Spain: with messaging

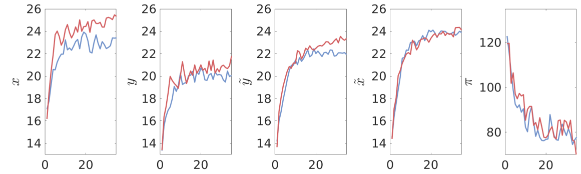

(e) China: no messaging

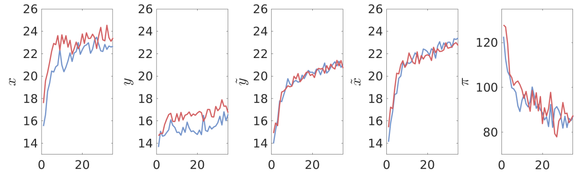

(f) China: with messaging

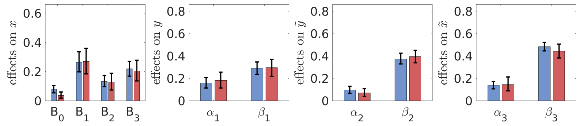

(g) China: no messaging

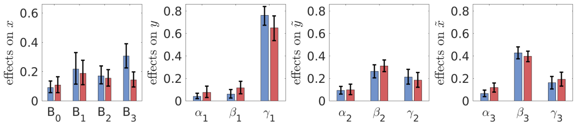

(h) China: with messaging

**Figure S15:** Differences in the dynamics of focal variables and parameters between individualist (red) and prosocial (blue) subjects. Parts (a-d) are reproduced from Figure S19 in Tverskoi *et al.* (2023b).

| Experiment | Mean | Median | St. dev | Skewness | Kurtosis |
|---|---|---|---|---|---|
| CPR-Spain | 0.57 | 0.68 | 0.39 | -0.32 | 1.56 |
| CPR-China | 0.62 | 0.65 | 0.35 | -0.33 | 1.66 |
| CR-2020 | 0.61 | 0.63 | 0.32 | -0.46 | 2.14 |

**Table S3:** Basic characteristics of the rule compliance rate distributions.

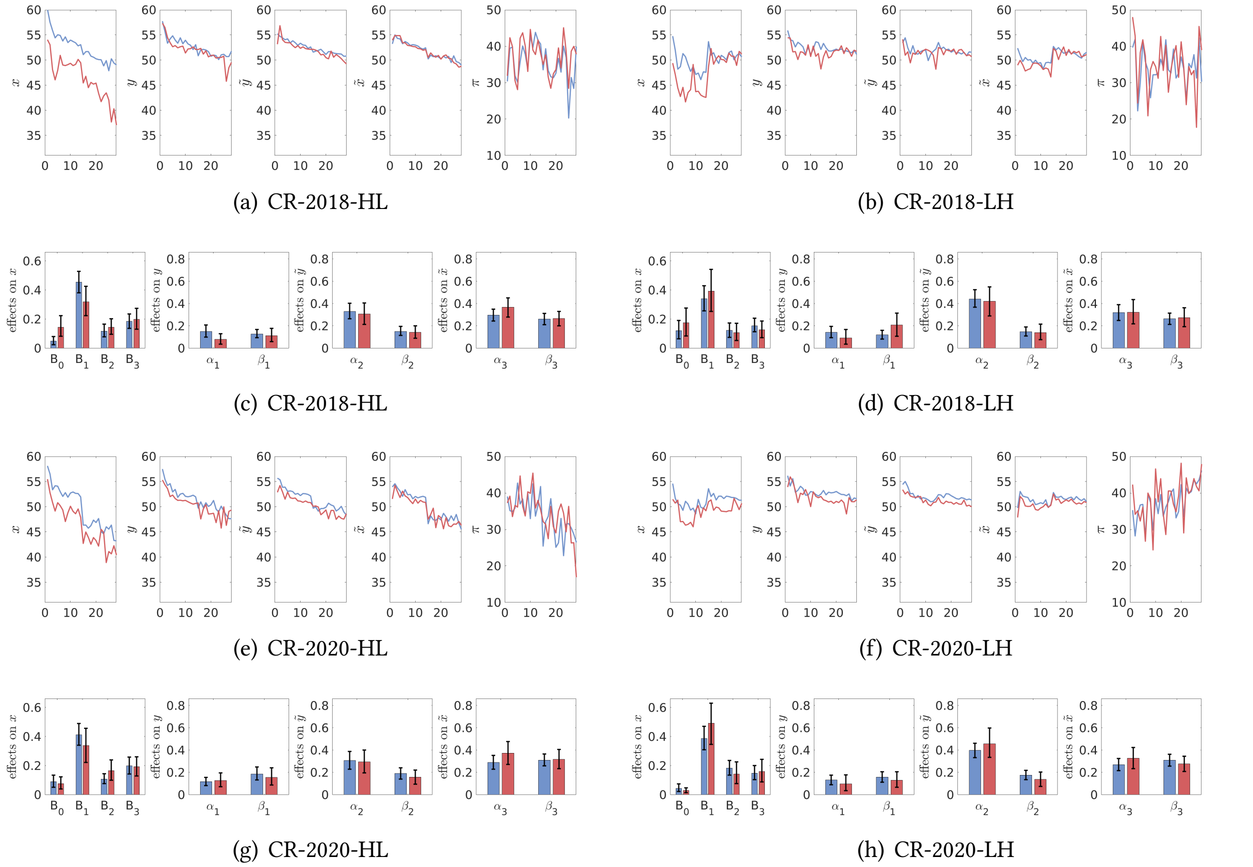


**Figure S16:** Differences in the dynamics of focal variables and parameters between individualist (red) and prosocial (blue) subjects.

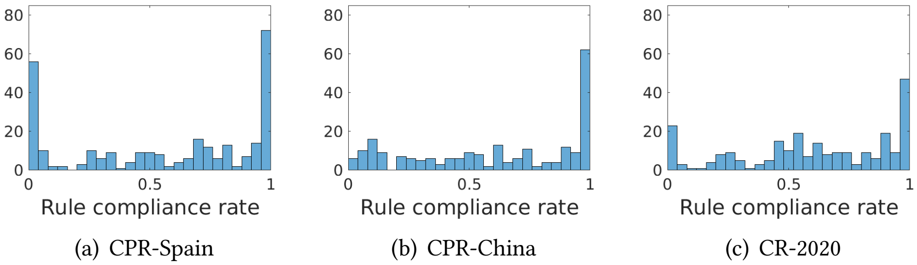


**Figure S17:** Histograms of the rule compliance rates from the ball task experiment.

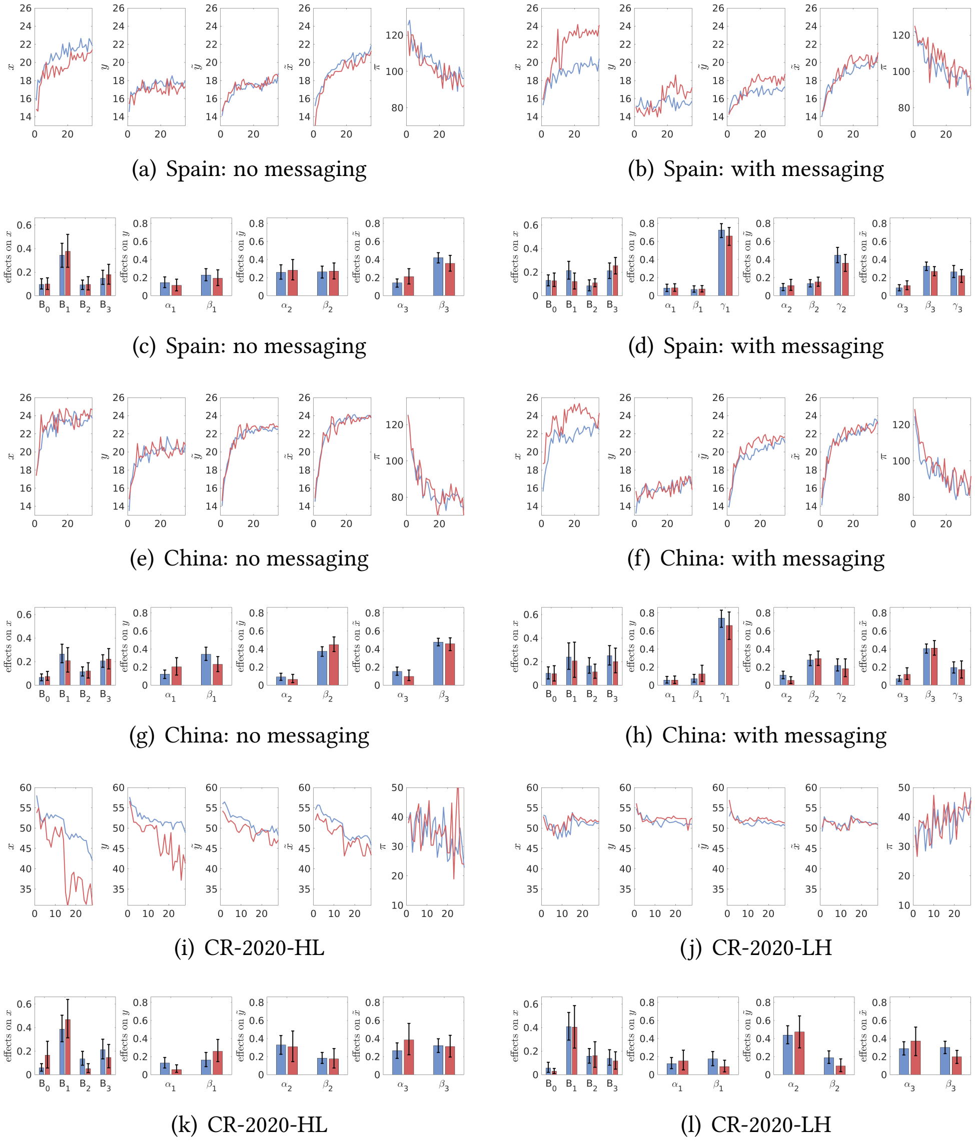


**Figure S18:** Differences in the dynamics of focal variables and parameters between rule-breakers (red) and rule-followers (blue). Parts (a-d) are reproduced from Figure S20 in Tverskoi *et al.* (2023b).

## S4.5 Comparing the results of cluster analysis, SVO tests, and rule-following tests

### S4.5.1 The relationship between prosociality and rule-following

As shown in Table S4, prosocial and rule-following tendencies are associated with each other in Spanish subjects, while no such association is observed in Chinese subjects. This can be another evidence of the different perception of prosociality between Spanish/Chinese subjects. Interestingly, in the CR-2020 experiment (which was conducted in the spring of 2020 during the lockdown in Spain), the share of individualist rule-breakers $(0.11)$ is much smaller while the share of prosocial rule-followers $(0.56)$ is much larger than in the CPR-Spain experiment (which was conducted in the spring of 2021 when COVID-19 restrictions were less strict) where these numbers are $0.25$ and $0.46$, respectively.

**Table S4:** Contingency table for the results of the SVO and rule-following tests in the CPR-Spain (first number), CPR-China (second number), and CR-2020 (third number) experiments. The corresponding associations are statistically significant for the CPR-Spain (odds ratio 5.0) and CR-2020 (odds ratio 2.5) subjects. Data on both treatments (with and without messaging in the CPR experiments and HL and LH risk levels in the CR-2020 experiments) are combined.

| | Prosocials | Individualists |
|---|---|---|
| Rule-followers | 93 / 58 / 79 | 28 / 44 / 19 |
| Rule-breakers | 33 / 30 / 27 | 50 / 23 / 16 |

### S4.5.2 The relationship between behavioral clusters, prosocial, and rule-following tendencies in the CPR experiments with messaging

**General results.** Since the differences between rule-followers/rule-breakers and prosocial/individualist types are quite more marked with messaging, here we focus only on the results of the CPR experiments with messaging. As discussed in the main body of the paper, we identified three types of subjects using parameters of the best response function: those whose decision-making is mostly driven by personal norms (Cluster 1), those with no single factor dominating in decision-making (Cluster 2), and those whose decision-making is mostly driven by conformity with others (Cluster 3, which was only identified in CPR-Spain). Here we show how this classification is related to the classifications based on the rule compliance and SVO tests. We will use two measures for each cluster: $\rho_{\text{RF}}$, which is the ratio of the number of rule-followers to the number of rule-breakers, and $\rho_{\text{PR}}$, which is the ratio of the number of prosocial subjects to the number of individualist subjects.

We put forward the hypothesis that subjects in Cluster 1 should have higher values of $\rho_{\text{RF}}$ and $\rho_{\text{PR}}$ compared to those in Cluster 2, who in turn should have higher values of $\rho_{\text{RF}}$ and $\rho_{\text{PR}}$ compared to subjects in Cluster 3. Table S5 shows that this hypothesis is mostly supported by our data: rule-followers and prosocial subjects are in significant majority in Cluster 1, in majority in Cluster 2, and in minority in Cluster 3. The only exception is that in CPR-China, $\rho_{\text{PR}}$ is closed to 1 in both presented clusters, which is not very surprising as the differences between prosocial and individualist subjects in terms of their actions (see Figure S18) are small. Overall, these results show that the behavioral types identified by clustering are consistent with the SVO and rule-following measures.

**More details on different perception of prosociality between Spanish/Chinese subjects.** Here we consider in more detail the differences between CPR-Spain and CPR-China in terms of distributions of prosocial and individualist subjects within our behavior clusters. To do this, we consider separately prosocial and individualist subjects in each cluster. We have three clusters in CPR-Spain and two clusters in CPR-China. Since Cluster 2 in the CPR-China can be treated as an analog to the union of Clusters 2 and 3 in the CPR-Spain, we merge the latter two clusters into one (which we will call Cluster 2) to conduct our comparative analysis. As a result, we have 4 groups of individuals: prosocial subjects of Cluster 1,

**Table S5:** Relationships between different classifications of subjects. Clusters 1-3 are defined on the basis of parameters of the best response function. $\rho_{RF}$ is defined as the ratio of the number of rule-followers to the number of rule-breakers in the cluster. $\rho_{PR}$ is defined as the ratio of the number of prosocial subjects to the number of individualist subjects in the cluster. For the rule-following measure, the numbers in parentheses are the proportions of subjects identified as either rule-followers or rule-breakers; the remaining subjects, which are neither rule-followers nor rule-breakers, are ignored.

| Experiment | Measure | Cluster 1 | Cluster 2 | Cluster 3 |
|---|---|---|---|---|
| CPR-Spain w/ messaging | size | 22 | 76 | 44 |
| | $\rho_{RF}$ | 3 (0.73) | 1.27 (0.77) | 0.84 (0.80) |
| | $\rho_{PR}$ | 4.5 | 1.30 | 1 |
| CPR-China w/ messaging | size | 20 | 83 | - |
| | $\rho_{RF}$ | 2.5 (0.70) | 1.85 (0.69) | - |
| | $\rho_{PR}$ | 1 | 1.18 | - |

prosocial subjects of Cluster 2, individualist subjects of Cluster 1, and individualist subjects of Cluster 2. The average trajectories and estimated parameters for these groups are shown in Figure S19.

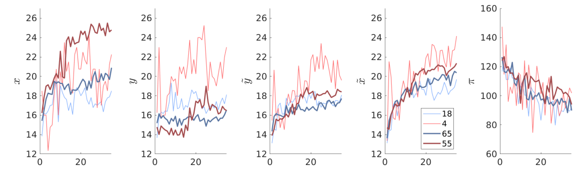

(a) Spain: with messaging

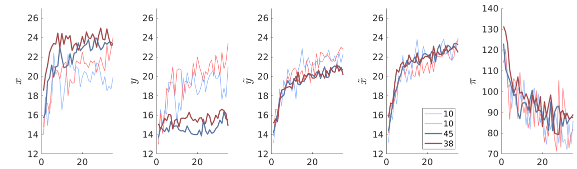

(b) China: with messaging

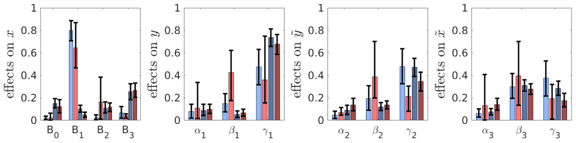

(c) Spain: with messaging

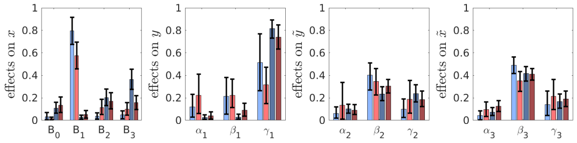

(d) China: with messaging

**Figure S19:** Differences in the dynamics of focal variables and parameters between individualist (red) and prosocial (blue) subjects. Subjects of Cluster 1 are colored light blue/red, while subjects of Cluster 2 are colored dark blue/red.

First, in both the experiments, actions are determined by personal norms of subjects of Cluster 1. For these subjects prosocial/individualist tendencies are expressed in terms of how personal norms are formed. For prosocial subjects, the effect of messaging (i.e., $\gamma_1$) dominates all other forces in the dynamics of personal beliefs, while for individualist subjects, other forces are as important as messaging in personal beliefs formation.

In both the experiments, individuals of Cluster 2 are those, whose decision making is affected by a combination of various factors. Moreover, these individuals do not use comprehensive methods for updating their personal norms so that they are very close to the value promoted by messaging (because they do not care much about personal norms, they can set them up in the simplest way). For these subjects prosocial/individualist tendencies are expressed in terms of coefficients of the best response function. Specifically, in CPR-Spain, prosociality is associated with higher weights $B_1$ of personal norms, while in CPR-China, prosociality is associated with higher weights $B_3$ of conformity (i.e., Spanish prosocial subjects are prone to act more in accordance with their perception of what is the right thing to do, while Chinese prosocial subjects are prone to conform with the "society").

### S4.5.3 The relationship between behavioral clusters, prosocial, and rule-following tendencies in the CR experiments

**General results.** As discussed in the main body of the paper, we identified three types of subjects using parameters of the best response function: those whose decision-making is mostly driven by personal norms (Cluster 1), those with no single factor dominating in decision-making (Cluster 2), and those whose decision-making is mostly driven by material factors (Cluster 3, which was only identified in CR-2018-LH). Here we show how this classification is related to the classifications based on the rule compliance and SVO tests.

We expected that in the HL treatment, where the differences between rule-followers/rule-breakers and prosocial/individualist subjects are quite pronounced, Cluster 1 would be characterized by higher values of $\rho_{\text{RF}}$ and $\rho_{\text{PR}}$ compared to Cluster 2; while in the LH treatment, where the corresponding differences are small, there would be not much difference between Clusters 1 and 2 in $\rho_{\text{RF}}$ and $\rho_{\text{PR}}$. Table S6 shows that our expectation is supported by data for prosocial/individualist subjects but not for rule-followers/rule-breakers. Specifically, $\rho_{\text{RF}}$ is higher in Cluster 1 than in Cluster 2 in the HL treatment, and $\rho_{\text{RF}}$ is higher in Cluster 2 than in Cluster 1 in the LH treatment.

**Table S6:** Relationships between different classifications of subjects. Clusters 1-3 are defined on the basis of parameters of the best response function. $\rho_{\text{RF}}$ is defined as the ratio of the number of rule-followers to the number of rule-breakers in the cluster. $\rho_{\text{PR}}$ is defined as the ratio of the number of prosocial subjects to the number of individualist subjects in the cluster. For the rule-following measure, the numbers in parentheses are the proportions of subjects identified as either rule-followers or rule-breakers; the remaining subjects, which are neither rule-followers nor rule-breakers, are ignored.

| Experiment | Measure | Cluster 1 | Cluster 2 | Cluster 3 |
|---|---|---|---|---|
| CR-2018-HL | size | 59 | 87 | - |
| | $\rho_{\text{PR}}$ | 3.92 | 1.49 | - |
| CR-2018-LH | size | 30 | 73 | 15 |
| | $\rho_{\text{PR}}$ | 2.33 | 2.43 | 1.5 |
| CR-2020-HL | size | 51 | 86 | - |
| | $\rho_{\text{RF}}$ | 1.55 (0.55) | 2.15 (0.48) | - |
| | $\rho_{\text{PR}}$ | 2.57 | 1.61 | - |
| CR-2020-LH | size | 50 | 90 | - |
| | $\rho_{\text{RF}}$ | 3.33 (0.52) | 2.54 (0.51) | - |
| | $\rho_{\text{PR}}$ | 2.57 | 2.9 | - |

**More details on different pathways to express rule-following tendency in the HL treatment.** Here we consider the differences in behavior of rule-followers and rule-breakers in the CR-2020 subjects in each behavioral cluster. First, as mentioned in the main text, the differences between rule-followers and rule-breakers are more pronounced in the HL experiment especially when the risk becomes low: rule-breakers tend to significantly reduce their actions compared to rule-followers. However, there are several ways to express the rule-following tendency (see Figure S20). First, for individuals in Cluster 1 (those, whose actions are mostly determined by their personal norms), the rule-following tendency is expressed in the way personal norms are defined. Specifically, personal norms of rule-breakers in Cluster 1 are mostly determined by actions of others, while for the rule-followers the effect of cognitive dissonance is also noticeable. As a consequence, rule-following individuals in Cluster 1 have higher personal norms and higher contributions (that do not exhibit a significant drop when the risk becomes low) among all the behavioral types. Second, for individuals in Cluster 2 (those, whose actions are determined by the effects of all factors), the rule-following tendency is expressed in terms of coefficients $B_0$, $B_2$, and $B_3$. Specifically, rule-breakers are characterized by a stronger effect of material factors (i.e., have higher $B_0$),

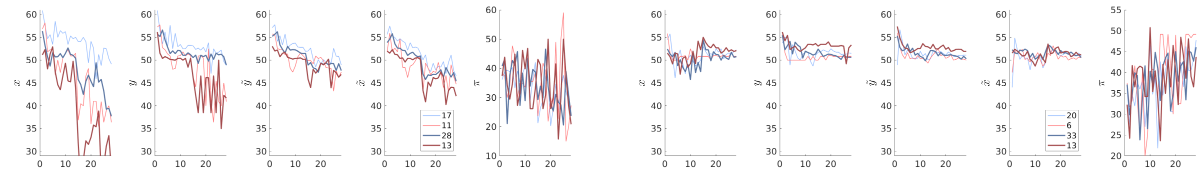

(a) CR-2020-HL (b) CR-2020-LH

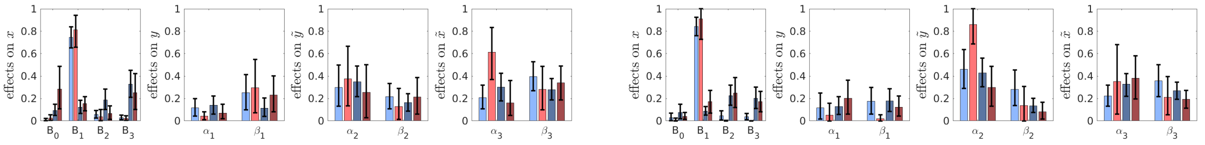

(c) CR-2020-HL (d) CR-2020-LH

**Figure S20:** Differences in the dynamics of focal variables and parameters between rule-followers (red) and rule-breakers (blue). Subjects of Cluster 1 are colored light blue/red, while subjects of Cluster 2 are colored dark blue/red.

while rule-followers tend to align their actions with the behavior of others (i.e., have higher $B_2$ and $B_3$).

### S4.5.4 The relationship between behavioral clusters, and clusters based on estimated coefficients of beliefs dynamics

In the CPR experiments with messaging, three behavioral clusters are identified: subjects whose decision-making is mostly driven by personal norms (Cluster 1); for subjects for whom no single factor dominates others in decision-making (Cluster 2); and subjects whose decision-making is mostly driven by conformity with others (Cluster 3, which is only present in the CPR-Spain). At the same time, there are two clusters based on estimated coefficients of beliefs dynamics: Cluster 1b represents subjects to whom the effects of cognitive forces, peer behavior, and messaging on personal norms are comparable in magnitude; while Cluster 2b represents individuals whose personal norms are mostly affected by messaging. Here, we look at the overlap between the two classifications. We expected that individuals in Cluster 1 would be more likely to be classified in Cluster 1b, while subjects in Clusters 2 and 3 would be more likely to be classified in Cluster 2b. Our expectation is supported by the results presented in Table S7. In fact, the data suggest that individuals who care little about their personal norms when making decisions, are more likely to adjust their personal norms in response to messaging (apparently because this is very easy to do); while those whose decision-making is mostly driven by personal norms, undergo a comprehensive personal norms adjustment based on cognitive dissonance, conformity with others, and messaging.

**Table S7:** The contingency table between behavioral clusters and clusters based on estimated coefficients of beliefs dynamics in the CPR experiments with messaging. In each cell, the first number is for the CPR-Spain and the second number is for the CPR-China. The corresponding associations are statistically significant in the CPR-Spain (odds ratio 3.5, we combined Clusters 2 and 3 to perform this analysis) and in the CPR-China (odds ratio 11.1).

| | Cluster 1b | Cluster 2b |
|---|---|---|
| Cluster 1 | 15 / 16 | 7 / 4 |
| Cluster 2 | 29 / 22 | 47 / 61 |
| Cluster 3 | 17 / - | 27 / - |

## S4.6 Stubborn individuals

We define an individual as a stubborn if they change their personal norm $y$ no more than twice over the course of the experiment. For such individuals, the values of parameters $\alpha_1$ and $\beta_1$ are expected to be small. Our results are shown in Figures S21 and S22 and Table S8.

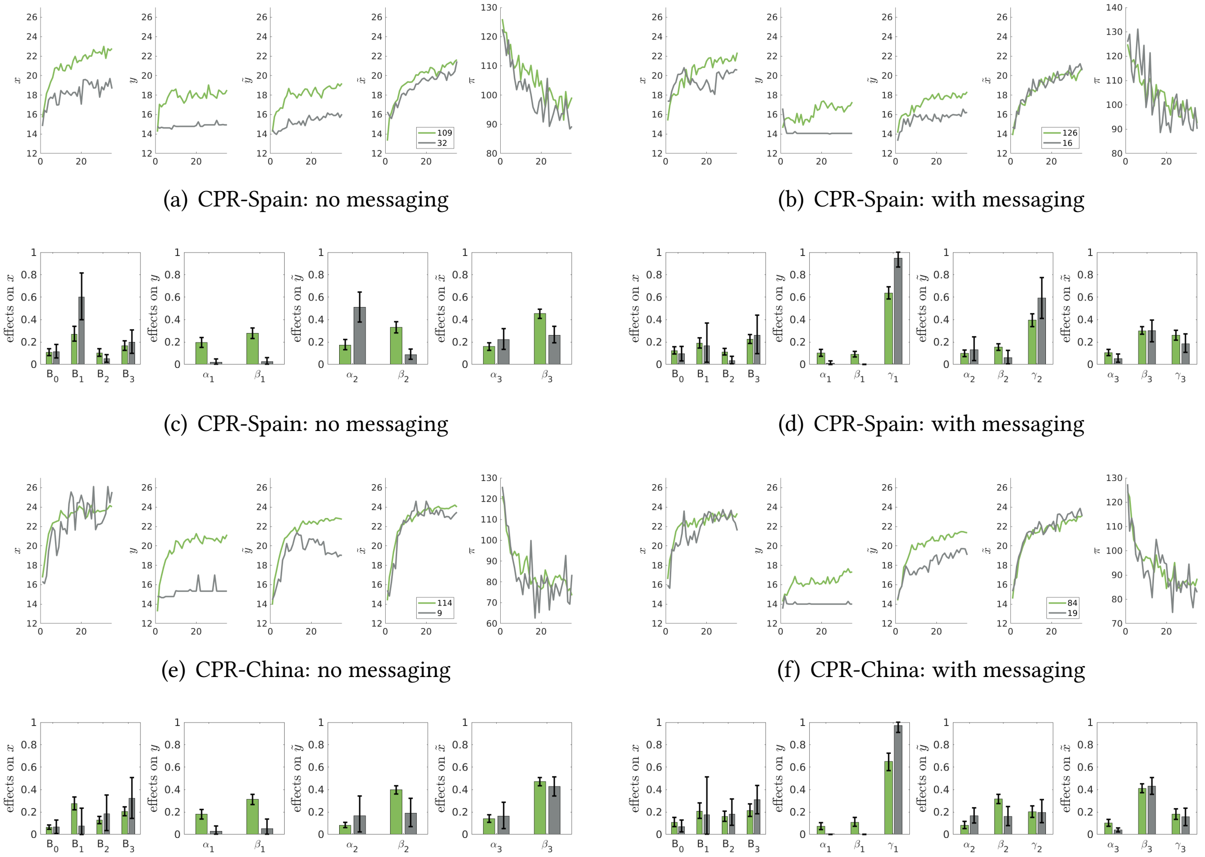


(a) CPR-Spain: no messaging
(b) CPR-Spain: with messaging
(c) CPR-Spain: no messaging
(d) CPR-Spain: with messaging
(e) CPR-China: no messaging
(f) CPR-China: with messaging
(g) CPR-China: no messaging
(h) CPR-China: with messaging

**Figure S21:** Differences in the dynamics of focal variables and parameters between stubborn individuals (black) and others (green) in the CPR experiments.

**CPR experiments without messaging**. There are 32 such individuals in the CPR-Spain and 9 in the CPR-China. In the CPR-Spain without messaging, stubborn individuals have larger $B_1$ and $\alpha_2$ and smaller $\beta_2$ and $\beta_3$ than other participants. As a result, they have smaller efforts $x$, normative $\tilde{y}$ and empirical $\tilde{x}$ expectations. In contrast, in the CPR-China without messaging, stubborn individuals have smaller $B_1$ compared to other participants. Their average contribution $x$ is similar to that of other individuals. Overall, in both CPR experiments without messaging, stubborn individuals have very low personal norms ($y = 15$). However, the main difference is that in the CPR-Spain personal norms play a key role in decision-making and belief formation of stubborn individuals, while in the CPR-China, stubborn individuals do not care too much about their personal norms, but put more weights on normative and empirical expectations in their decision-making. Note that there are more stubborn individuals in the CPR-Spain (32) than in the CPR-China (9).

**CPR experiments with messaging**. There are 16 such individuals in the CPR-Spain and 19 in the CPR-China. In these experiments, personal norms $y$ of stubborn individuals are fixed at the level promoted

by messaging. Also, stubborn individuals have lower normative expectations $\tilde{y}$ compared to other subjects, and, in the CPR-Spain, make slightly smaller efforts $x$. They are also characterized by larger $\gamma_1$ compared to other participants. In the CPR-Spain, stubborn individuals have larger $\gamma_2$ and smaller $B_2$ compared to other participants. The main difference between two experiments is that in the CPR-China stubborn individuals put more weights on normative beliefs which are larger due to significantly smaller effect of messaging.

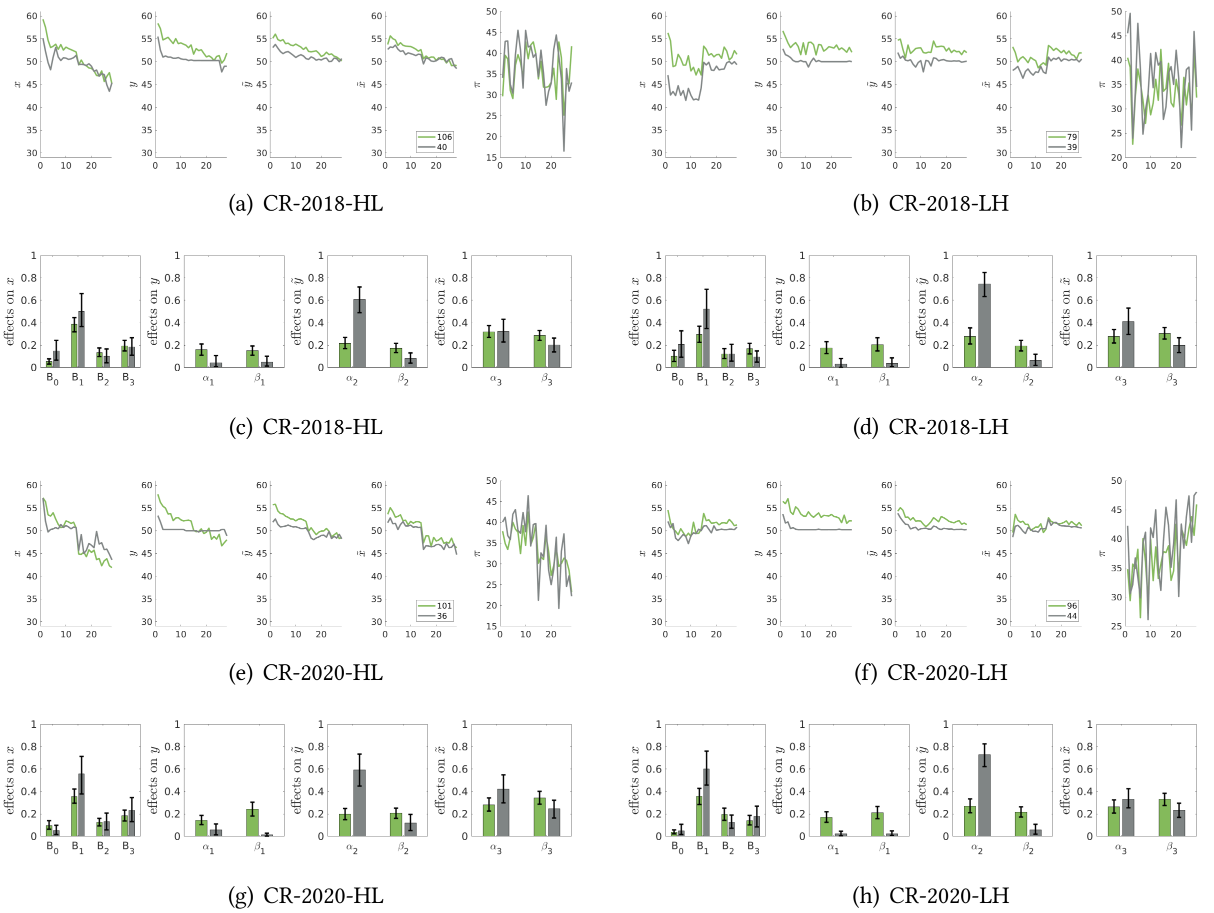

(a) CR-2018-HL (b) CR-2018-LH (c) CR-2018-HL (d) CR-2018-LH (e) CR-2020-HL (f) CR-2020-LH (g) CR-2020-HL (h) CR-2020-LH

**Figure S22:** Differences in the dynamics of focal variables and parameters between stubborn individuals (black) and others (green) in the CR experiments.

**CR experiments**. There are 79 such individuals in the CR-2018 and 80 in the CR-2020. In these experiments, stubborn individuals have personal norms close to $y = 50$ which are typically smaller that those of other individuals. Typically, they make smaller contributions $x$, and their normative expectations are close to personal norms. This is because they are characterized by larger effects of personal norms on contributions and normative expectations (i.e., have larger $B_1$ and $\alpha_2$). Overall, stubborn individuals in the CR experiments are those who believe this is the right thing to make a fair contribution of $x = 50$ and they incorporate this belief in their decision-making and the formation of normative expectations. Interestingly, stubborn individuals are characterized by a larger effect of material factors on their decision-making compared to other participants in CR-2018 experiments. This phenomenon is not observed in CR-2020 experiments. Perhaps, this is the result of Covid-19 pandemic or other external factors.

In the CR-experiments, the number of stubborn individuals (about 40 per treatment) is sufficient for additional statistical analysis. The results (see Table S8) show that the share of Cluster 1 individuals among

stubborn subjects is the same as in the entire population (except, CR-2018-LH experiment). Intuitively, prosocial individuals are less common among stubborn individuals than in the entire group. The same result is observed for rule-followers but only in the HL treatment when the rule is more clear (i.e., to keep making large contributions after the probability of disaster decreases).

| Experiment | total # | $r_{Cl1}$ | $r_{PR}$ | $r_{RF}$ |
|---|---|---|---|---|
| CR-2018-HL | 40 | 0.38 (0.40) | 0.60 (0.68) | - (-) |
| CR-2018-LH | 39 | 0.33 (0.25) | 0.59 (0.69) | - (-) |
| CR-2020-HL | 36 | 0.39 (0.37) | 0.58 (0.65) | 0.45 (0.65) |
| CR-2020-LH | 44 | 0.39 (0.36) | 0.68 (0.74) | 0.72 (0.74) |

**Table S8:** The relationship between stubborn individuals and different classifications of individual types. Shown are: (1) the total number of stubborn individuals in each experiment (total #); (2) the share of individuals belonging to behavioral Cluster 1 ($r_{Cl1}$); (3) the share of prosocial subjects ($r_{PR}$); and (4) the share of rule-following subjects ($r_{RF}$). The corresponding shares are calculated among stubborn individuals and in the entire population (shown in parentheses).

## S4.7 Conditional compliers

Our earlier work (Tverskoi *et al.*, 2023b) identified a new class of individuals who comply with messaging as long as they see others are complying. We called them "conditional compliers" by analogy with "conditional cooperators" (Andreozzi *et al.*, 2020; Fischbacher and Gächter, 2010) who cooperate if they believe others will be cooperating. For conditional compliers the dynamics of efforts in the CPR game was described by an S-shaped function with relatively low efforts initially and a sharp transition to relatively high efforts in the middle of the the experiment when the average contributions of others exceed a certain threshold. The behavior of conditional compliers was not well described by our linear best response function (2) leading to some mismatch between the observed and predicted average trajectories (Figures S9-S10).

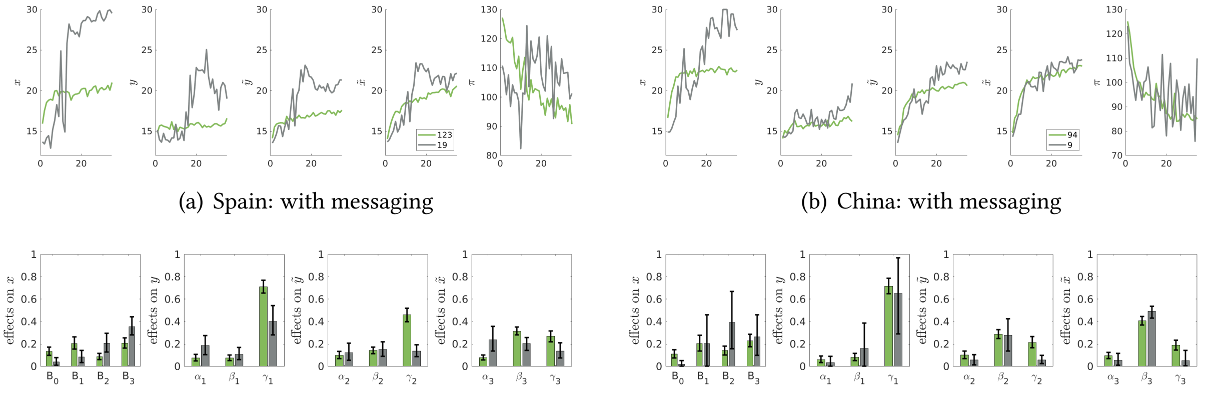


(a) Spain: with messaging

(b) China: with messaging

(c) Spain: with messaging

(d) China: with messaging

**Figure S23:** Differences in the dynamics of focal variables and parameters between conditional compliers (black) and others (green).

Here we say that an individual is a conditional complier if their average effort $x$ in the first 8 rounds is below the population average but the average effort in the last 8 rounds is 20% higher than the population average. There are 19 conditional compliers in the CPR-Spain and 9 in the CPR-China. As shown in Figure S23 in their decision-making, conditional compliers put more weight on behavior of others $B_2+B_3$,

lower weight of material factors $B_0$, and lower effects of messaging $\gamma_i$ on the dynamics of beliefs (except for the case of personal norms, $\gamma_1$, in the CPR-China). Note also that when conditional compliers are excluded, the trajectory of the average value of $y$ becomes much smoother and the step-like increase in the middle of the CPR-Spain experiment with messaging disappears.

It may seem counter-intuitive that conditional compliers are characterized by a relatively small effect of material factors on their decision making, but it makes sense. Indeed, an individual maximizing their material payoff should have large contributions when the contribution of others is small; and reduce the contribution later on when the average contribution of others increases. This is the exact opposite of the behavior of conditional compliers.

## S4.8 Individual types, cooperation, and the effect of messaging

Above we have classified individuals to several types according to different criteria, including two/three clusters of individuals based on coefficients $B_i$, two types of individuals (prosocial and individualists) based on the SVO analysis, and two types of individuals (rule-followers and rule-breakers) based on the rule following analysis. In addition, we have also identified two specific types of individuals, stubborn individuals and conditional compliers. Figure S24 illustrates which individuals are more important in promoting cooperation and, in the CPR case, are more sensitive to messaging. Note that different classifications may overlap and the same individuals can belong to different characters.

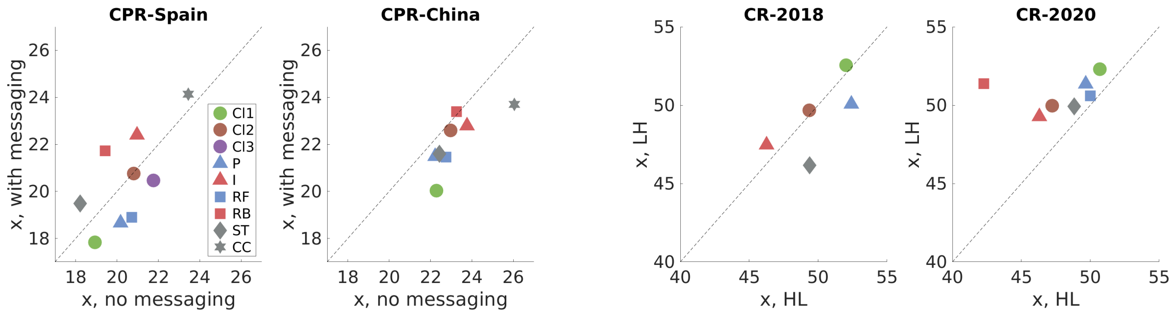


(a) CPR experiments (b) CR experiments

**Figure S24:** The average contributions of various individual types under the two treatments. (a) The CPR experiments without and with messaging in the CPR-Spain (left) and CPR-China (right) (b) The CR experiments under HL and LH treatments in CR-2018 (left) and CR-2020 (right) experiments. Different symbols show: individuals belonging to behavioral Cluster 1 (Cl1); individuals belonging to behavioral Cluster 2 (Cl2); individuals belonging to behavioral Cluster 3 (Cl3); prosocial subjects (P); individualist subjects (I); rule-followers (RF); rule-breakers (RB); (8) Stubborn individuals (ST); and (9) Conditional compliers (CC). In the CPR experiments, types closer to the lower left corner can be treated as more cooperative; while types closer to the upper right corner can be treated as less cooperative. Types that are below the diagonal can be treated as those who reduce their efforts in response to messaging, while types that are above the line can be treated as those who increase their efforts in response to messaging. In the CR experiments, types closer to the lower left corner can be treated as less cooperative; while types closer to the upper right corner can be treated as more cooperative. Types that are below the diaginal can be treated as those who reduce their efforts under the HL treatment compared to the LH treatment, while types that are above the line can be treated as those who increase their efforts under the HL treatment compared to the LH treatment.

**The CPR experiments**. In both experiments without messaging, Cluster 1 individuals and stubborn individuals are the most cooperative individual types (i.e., make the smallest extraction efforts), while conditional compliers are the least cooperative type (i.e., make the largest extraction efforts). This makes sense because decision-making of Cluster 1 and stubborn individuals is mostly determined by personal norms. The latter capture an individual's perception of what is the right thing to do, which is typically to contribute less. Conversely, conditional compliers make the largest efforts at the end of the experiments. With messaging, Cluster 1 individuals, stubborn individuals, prosocial participants, and rule-followers

are among the most cooperative types; while conditional compliers, individualist participants, and rule-breakers are among the least cooperative types. In both experiments, messaging has the highest effect on prosocial and rule-following types as well as on individuals from Cluster 1. Individuals from Cluster 2 are not affected by messaging. In the CPR-Spain, messaging reduces effort of individuals from Cluster 3, but backfires in individualists, rule-breakers and stubborn subjects. In the CPR-China, messaging also reduces effort of stubborn subjects and individualists.

**The CR experiments**. In all experiments, individualist subjects are among the least cooperative types (i.e., make relatively small contributions $x$), while Cluster 1 individuals and prosocial subjects are among the most cooperative types (i.e., make relatively large contributions $x$). Rule-breakers exhibit the least cooperative behavior among all types but only in the HL treatment. This is likely because a clear "rule" emerges only during the period when the probability of disaster is very large and the subject follow this rule by behavioral inertia even after the risk reduces.

## S4.9 Gender differences

In the CPR-China experiment with messaging, females extract more resources than males and have higher values of $y, \tilde{y}$ and $\tilde{x}$ (see Figure S25 in SM). They also pay more attention to the behavior of others when updating beliefs (they have larger values of parameters $\beta_i$). In the three other CPR experiments, there is no difference between average extraction of the sexes. Relative to males, personal norms of females are larger in the CPR-Spain without messaging but smaller in CPR-China with messaging. Other differences in parameters are either not significant or specific to a particular experiment.

In the CR experiments, males generally contribute less than females, with this effect being more pronounced under high risk (see Figure S26 in SM). This is likely due to males having large weights of material payoffs ($B_0$) although only in one treatment (CR-2018-LH) the difference is statistically significant. In three out of four cases, males have larger $\alpha_3$ (logic constraints). Other differences in parameters are either not significant or specific to a particular experiment.

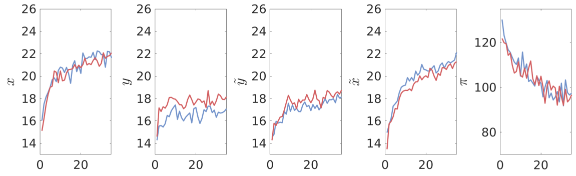
(a) Spain: no messaging

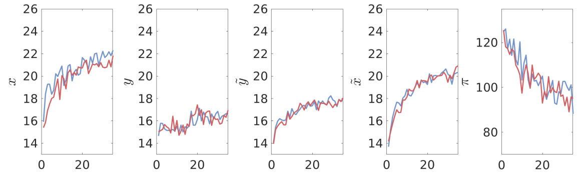
(b) Spain: with messaging

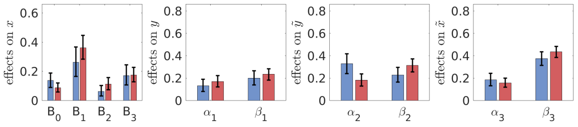
(c) Spain: no messaging

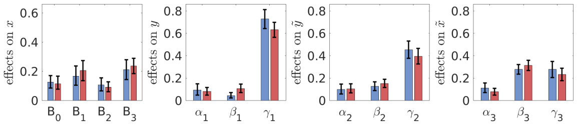
(d) Spain: with messaging

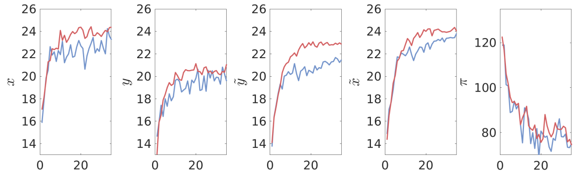
(e) China: no messaging

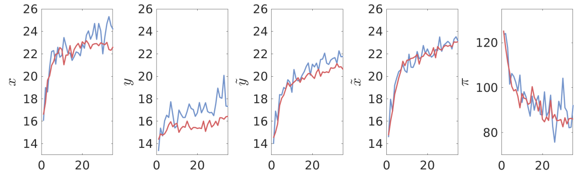
(f) China: with messaging

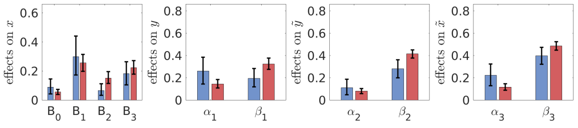
(g) China: no messaging

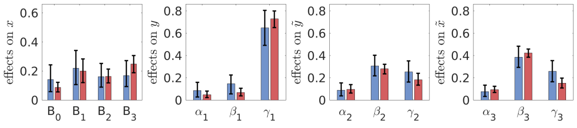
(h) China: with messaging

**Figure S25:** Differences in the dynamics of focal variables and parameters between females (red) and males (blue). Parts (a-d) are reproduced from Figure S18 in Tverskoi *et al.* (2023b).

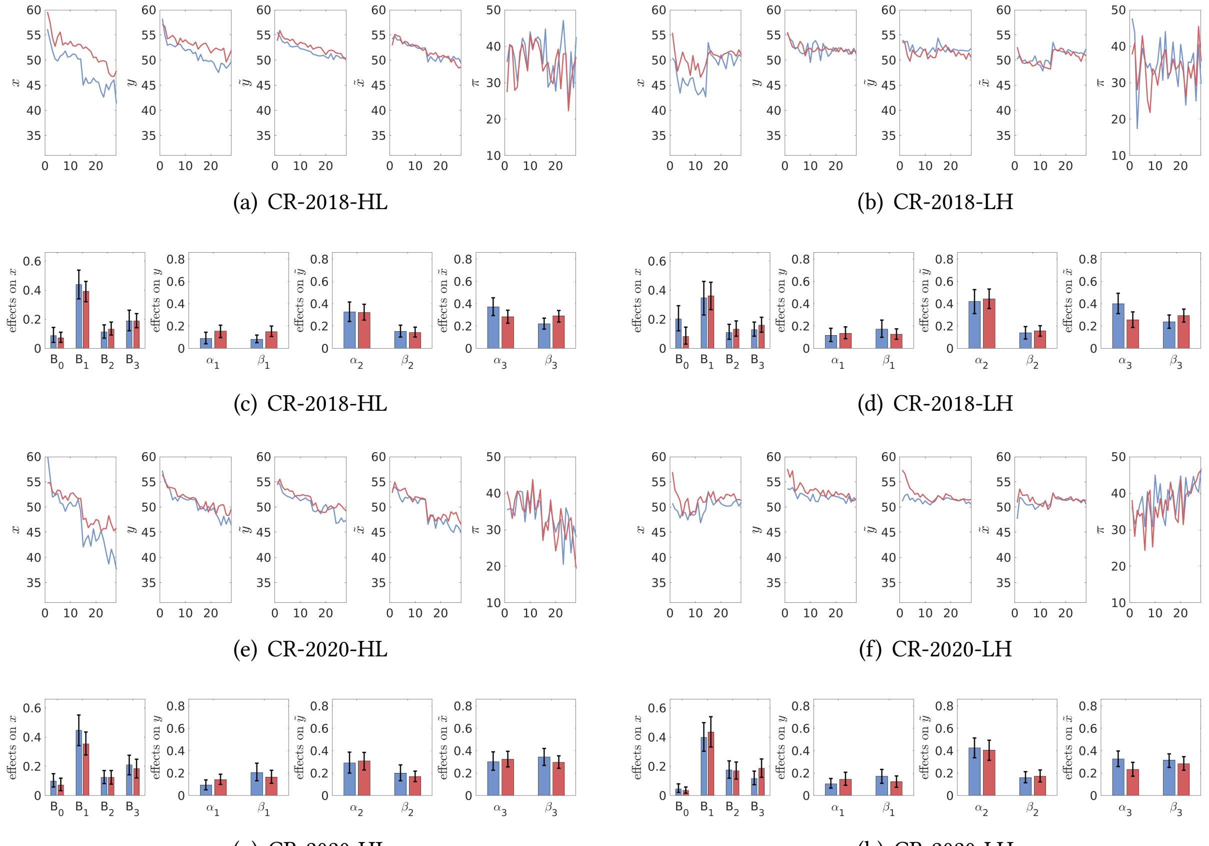

(a) CR-2018-HL (b) CR-2018-LH

(c) CR-2018-HL (d) CR-2018-LH

(e) CR-2020-HL (f) CR-2020-LH

(g) CR-2020-HL (h) CR-2020-LH

**Figure S26:** Differences in the dynamics of focal variables and parameters between females (red) and males (blue).

# S5. Instructions to subjects.

Starting on the next page we give an English translation of the instructions to the subjects in the Common Pool Resources game. An English translation of the instructions given to the subjects in the Collective Risk experiments is available from the Supplementary Information in Szekely *et al.* (2021).

## S5. Instructions to subjects (in English)

### Welcome screen

Welcome and thank you for participating in this experiment. The experiment consists of seven parts that will take place over 36 days. You will start with the first five parts, which will take approximately 20 minutes.

Please do so as soon as you read these instructions. These parts are mandatory and if you do not complete them you will be excluded from the experiment. You have until 10:00 a.m. CEST tomorrow morning to complete the first five parts. After that, you will move on to part six. He will make his first Part 6 decision tomorrow.

Thereafter, you will participate in a new round of part 6 on the remaining days. Each day you will have until 10 a.m. CEST the following day to make your decision. Your decisions in this part will take you only a few minutes per day.

Finally, you will reach the last part, the seventh part.

### Important rules

Your participation is voluntary and you may withdraw from the experiment at any time. However, if you withdraw you will not receive any payment.

We strongly ask you not to communicate with any other participant and, in general, to make your decisions independently. Please do not share your personal participation link with anyone.

Your responses must be kept confidential. The researchers who will analyze the experiment data will not be able to link your identity to your decisions in any way.

Throughout the experiment you will not be lied to or misled in any way: what we say, we do.

### Payment

You will be paid at the end of the experiment via PayPal. Your payment will be the sum of all your earnings from each part. For every 10 tokens you earn you will receive 1 euro. Your earnings will depend partly on your decisions and partly on those of other participants. You will be paid directly into your PayPal account so that the other participants will not be able to know how much you have won. You will be given additional instructions about your earnings at a later date.

If you do not complete today's decisions or fail to make 3 decisions during the experiment, you will be automatically and permanently expelled from the experiment and, in that case, you will not receive any payment.

At the end of the experiment, 3 participants will be randomly selected from those who have completed all parts of the experiment. Those selected will receive an additional payment, consisting of a 10-fold increase in their earnings. We guarantee that this randomly selected person will win at least 100 euros, to be added to the rest of their payouts.

**Additional information**

Once you click on the "Next" button you will not be able to return to the previous screen, so please read the instructions carefully. In any case, we will show you relevant instructions during the experiment. If you have any questions during the experiment, please write to ibsen.gisc@gmail.com. When you are ready to continue, click on "Next" below.

*[After this page participants perform Big 5 (section 1), SVO test (section 2), Autism spectrum (section 3), demographic questions (section 4) and risk preference test (section 5)]*

***End of day 1 page***

You have completed all decisions of today. Come back tomorrow at 10 AM (Madrid time).

***Instructions for section 6***

You are beginning the sixth part of the experiment. Please read the instructions for this part below.

**Instructions**

Groups and rounds. You will be making decisions for 35 days. Each day, you will be randomly included in a group of 6 participants including yourself, all randomly selected. The composition of the group will change every day, so each day you could potentially be grouped with five new people. You will not be able to know who the other 5 participants are.

**How to make decisions**: Each round you will be given an allocation of 30 tokens. All members of your group will receive the same 30 token allocation. In each round you

will have to decide how to use your tokens. You will make your decisions individually. All group members must make their decisions at the same time, i.e. during the same day.

**Common Account:** You can place in the Common Account any whole number of tokens between 0 and 30. The tokens in your group's Common Account will be multiplied by a certain factor and distributed among the 6 members of the group (including you) in proportion to the amount each participant has put in.

The earnings of the Common Account decrease with the total amount of tokens put into it by your group. This means that the earnings you get from the pool depend not only on the amount of tokens you decide to put into it but also on how much you put in between all the members of the group in each round. More information on the formula used to calculate the earnings will be shown below.

**Personal Account**: The tokens you have left after the allocation to the Common Account are for you.

**Total earnings in each round**: Your total earnings in a round are given by the sum of the tokens kept for you plus your earnings from the Common Pool.

To facilitate your decision, you will have access to an interactive tool when you go to make your decision, and to a complete table indicating your earnings in each case according to what you allocate to the Common Account (X) and what the other members of your group do (Y). To see the table now, click on the following link.

**Information in each round**: After each round, before making a new allocation decision, you will be given information on the decisions of the other members of your group. So, in each period, after everyone has made their decisions you will be able to see:

- Your own decision in that round
- The allocation decisions to the Pooled Account by the other members of your group in the previous round
- Your earnings from the Pooled Account in the previous round
- The amount of tokens you allocated to your Personal Account in the previous round
- The sum of your earnings from the Joint and Personal Accounts in the previous round

**Final earnings of the sixth part:** At the end of the experiment the system will randomly select 5 rounds, one of the first 7, one of the next 7, and so on. The amount you have won in those five particular decisions will be added to your final earnings. Each round is independent of the others as far as earnings are concerned.

**Additional questions and tokens**: Before each decision you will be asked some additional questions that will allow you to earn extra tokens. Please read the instructions on the screen carefully before making each decision.

At the end of the experiment, 3 participants will be randomly selected from those who have completed all parts of the experiment. Those selected will receive additional earnings, consisting of their earnings being multiplied by 10. We guarantee that this randomly selected person will win at least 100 euros, to be added to the rest of their earnings.

Remember, if you fail to make 5 decisions during the experiment you will be permanently and automatically excluded from the experiment. If a person does not make his/her decision in a round he/she will be assigned a decision from one of the other members of his/her group chosen at random.

Write to ibsen.gisc@gmail.com if you have any questions.

You will find these instructions at the bottom of each page. Here are some examples to make sure you understand everything correctly.

***Examples screens***

Remember that in each round you will be grouped with five other participants. Each participant receives 30 points in each round. Below are examples of situations that may occur during the experiment. The calculations in each example refer to the box in the table that indicates the corresponding winnings. You can see the winnings table here.

*Example 1*

You put 0 points in the Common Account and the other five participants also put 0 points. Therefore, the total amount in the Joint Account is 0 points. The amount you have left in your Personal Account is 30 points (30 - 0). Your final earnings are:

Your earnings from the Common Account: 0 points

Your earnings from your Personal Account: 30 points

Your total earnings in the round (Personal + Common): 30 points (note the box in the first row and first column of the table).

*Example 2*

You put 0 points in the Common Account and the other five participants also put 150 points between them (30 each). Therefore, the total amount in the Common Account is 150 points. The amount you have left in your Personal Account is 30 points (30 - 0). Your final earnings are:

Your earnings from the Common Account: 0 points

Your winnings from your personal account: 30 points

Your total earnings in the round (personal + common): 30 points (note the box in the last row and first column of the table).

*Example 3*

You put 30 points in the Common Account and the other five participants put 0 points between them. Therefore, the total amount in the Common Account is 30 points. The amount you have left in your personal account is 0 points (30 - 30). Your final earnings are:

Your earnings from the Common Account: 375.3 points.

Your earnings from your personal account: 375.3 points

Your total earnings in the round (Personal + Common): 30 points (note the box in the first row and last column of the table).

*Example 4*

You put 30 points in the Common Account and the other five participants also put 150 points between them (30 each). Therefore, the total amount in the Common Account is 180 points. The amount you have left in your personal account is 0 points (30 - 30). Your final earnings are:

Your earnings from the joint account: 1.8 points.

Your earnings from your personal account: 0 points

Your total earnings in the round (personal + common): 1.8 points (note the box in the last row and last column of the table).

## ***<u>Questionnaire screens</u>***

Please answer the questions we ask you below. We do this to help you better understand part 6. Your answers here do not affect your payments at all. Remember, you have the complete experiment instructions in the box at the bottom of this screen (scroll down if you do not see it).

**Question 1:**

If you put 5 points into the Common Account, and the other participants put in 0 points between them all:

How much do you earn from your Personal Account?

How much are your total earnings from both the Common and Personal Accounts?

**Question 2:**

If you put 5 points into the Common Account, and the other participants put 150 points between them all:

**Question 3:**

If you put 28 points into the Common Account, and the other participants put 150 points between them all:

**Question 4:**

If you put 28 points in the Common Account, and the other participants put 5 points between all:

**Question 5:**

Select the correct option:

If you fail to make 3 decisions during the experiment:

- You will be able to continue participating in the experiment without any problem.
- You will be automatically and permanently expelled from the experiment and, in that case, you will not receive any payment.

[*after responding to the questionnaire, subjects see a page with correct answers and explanation for wrong answers*]

***Beginning of the round screens***

You are going to start round X of 35. This means that you are going to participate in day X+1 of 37 of the experiment.

In this round, you have been randomly grouped with five other participants.

These participants may be different from those you were with on previous days.

Click "Next" to start today's round.

*[only for treatment with messaging]*

***Message screen***

Important message

Please note that the total group profit is maximized if each player contributes 14 points to the Common Account.

Note: this message is being communicated to all participants in the experiment.

***Belief elicitation screens***

*[Screen 1: Personal Normative Beliefs]*

Additional questions

In your opinion, how many points should a participant from your group, including yourself, put into the Common Account in this round?

*[Screen 2: Empirical expectations]*

You now have the opportunity to earn additional points. You will be informed at the end of the experiment whether you have earned them or not.

How many points will the other five participants in your group put into the Common Account in this round?

Use the boxes shown below to indicate how many points you think the other participants in your group will put into the Common Account this round. Please put the highest value in the top box and then order the amounts you respond with from highest to lowest. You may repeat amounts if you like, and in that case the order does not matter. We will order what the other participants in your group put in this round and compare with your answer.

For each answer that exactly matches one of yours you will receive 5 points. This means that you can earn a maximum of 25 points. The less closely your answer matches the decision of the other participants, the fewer points you will receive. If your answer differs from the true values by more than 5 you will receive 0 points. For example, if you believe that one of the other participants in your group will allocate X points to the Common Account, and that participant actually contributes X points, you will earn an additional 5 points for that answer.

*[Screen 3: Normative expectations]*

You now have the opportunity to earn additional points. You will be informed at the end of the experiment whether you have earned them or not.

How many points do the other 5 participants in your group that you should put into the Common Account in this round?

Use the boxes shown below to indicate how much you think the other participants in your group think you should allocate to the Common Account in this round. Please put the highest value in the top box and then order the amounts you respond with from highest to lowest. You may repeat amounts if you like, and in that case the order does not matter. We will order what the other participants in your group answer in this round and compare with your answer.

For each answer that exactly matches one of yours you will receive 5 points. This means that you can earn a maximum of 25 points. The less closely your answer matches the

belief of the other participants, the fewer points you will receive. If your answer differs from the true values by more than 5 you will receive 0 points. For example, if you believe that one of the other participants in the group answered with X points to the question "How much should each participant allocate to the Common Account?" that was shown to you two screens ago, and that participant's answer is X points, you will earn 5 additional points for that answer.

***Decision screen***

It is time to decide how to distribute your points.

How many points do you want to put into the Common Account in this round?

You can make a simulation of your earnings depending on your decision and the decision of the other 5 participants in the group in this round below.

Click "Next" to confirm your decision.

*[here is the slider widget for simulating payoffs]*

Your contribution:

Total contribution of the others:

Your earnings:

[*here is displayed the link to the pdf table summarizing all possible payoffs]*

Click here to view the pdf file of the winnings table.

***End of the round screen***

End of round

You have completed all your decisions for today. Come back tomorrow at 10:00 a.m. Spanish Summer Time (CEST).

***Results of the previous round***

**Part 6: round X**

You received an allocation of 30 points.

You allocated ___ to the Common Account and your group put in a total of ___.

The complete list of points allocated to the Common Account by your group is shown below. The choices of others are shown in random order.

Click "Next" to continue.

***Part 7 Punishment strategy task***

You have been randomly paired with another participant in the experiment. That other person is someone you do not know, nor does she know who you are. All your decisions will be confidential.

All participants, including you, receive 30 points.

You will be shown 6 possible distributions of your points (e.g., 0 to 5, 6 to 10, ..., 30) that the person you have been matched with could have chosen in round 35 of Part 6.

For each possible range of decisions, you will have to decide whether or not you want to spend some of your points to subtract points from the earnings of the person you have been matched with. Therefore, you will make 6 decisions.

For each decision slot, you can spend up to 10 of your points. Each point you spend will reduce the other person's points by 3 points. That is, if you spend 10 points you will subtract 30 points from the person you are matched with.

The other person you are matched with will also decide whether or not to reduce their earnings from you based on your decision in the 35 round of Part 6.

At the end of the experiment, the computer randomly selects whether to apply your decision and subtract points from the other person or to apply the other person's choice and subtract points from you.

The other person's points will be reduced based on what you decided here and what the other person would have decided in the 35 round of Part 6. For example, if the person you were paired with allocated X points, your points will be reduced by the amount you decided to allocate to that decision.

If you don't make your decisions here, you will automatically earn nothing for this part.

*Decision page*

How many points do you want to allocate to reduce the earnings of the person you have been matched with if he/she put into the pool

0 points?

1 to 5 points?

From 6 to 10 points?

…

From 26 to 30 points?

## References


Akerlof, G. A. and Dickens, W. T. (1982). The economic consequences of cognitive dissonance. *The American Economic Review*, **72**, 307–319.

Andreozzi, L., Ploner, M., and Saral, A. S. (2020). The stability of conditional cooperation: beliefs alone cannot explain the decline of cooperation in social dilemmas. *Scientific Reports*, **10**, 13610.

Calabuig, V., Olcina, G., and Panebianco, F. (2018). Culture and team production. *Journal of Economic Behavior and Organization*, **149**, 32–45.

Fischbacher, U. and Gächter, S. (2010). Social preferences, beliefs, and the dynamics of free riding in public goods experiments. *American Economics Reviews*, **100**, 541–556.

Gavrilets, S. (2021). Coevolution of actions, personal norms, and beliefs about others in1social dilemmas. *Evolutionary Human Sciences*, **3**, e44.

Kimbrough, E. O. and Vostroknutov, A. (2016). Norms make preferences social. *Journal of the European Economic Association*, **14**, 608–638.

Kimbrough, E. O. and Vostroknutov, A. (2018). A portable method of eliciting respect for social norms. *Economics Letters*, **168**, 147–150.

Kuran, T. and Sandholm, W. H. (2008). Cultural integration and its discontents. *Review of Economic Studies*, **75**(1), 201–228.

MacQueen, J. (1967). Classification and analysis of multivariate observations. In *5th Berkeley Symp. Math. Statist. Probability*, pages 281–297. University of California Los Angeles LA USA.

Murphy, R. O. and Ackermann, K. A. (2014). ocial value orientation theoretical and measurement issues in the study of social preferences. *Pers Soc Psychol Rev*, **18**, 13–41.

Murphy, R. O., Ackerman, K. A., and Handgraaf, M. J. J. (2011). Measuring social value orientation. *Judgment and Decision Making*, **6**, 771–781.

Rabin, M. (1994). Cognitive dissonance and social change. *Journal of Economic Behavior and Organization*, **24**, 177–194.

Szekely, A., Lipari, F., Antonioni, A., Paolucci, M., Sánchez, A., Tummolini, L., and Andrighetto, G. (2021). Collective risks change social norms and promote cooperation: Evidence from a long-term experiment. *Nature Communications*, **12**, 5452.

Tverskoi, D., nd Giulia Andrighetto, A. G., Sánchez, A., and Gavrilets, S. (2023a). Disentangling material, social, and cognitive determinants of1human behavior and beliefs. *Humanities and Social Sciences Communications*, **0**, 0–0.

Tverskoi, D., Guido, A., Andrighetto, G., Sánchez, A., and Gavrilets, S. (2023b). Disentangling material, social, and cognitive determinants of1human behavior and beliefs. *Humanities and Social Sciences Communications*, **0**, 0–0.

Vriens, E., Szekely, A., Lipari, F., Antonioni, A., Sánchez, A., Tummolini, L., and Andrighetto, G. (2023). Can pandemic risk promote cooperation and social norms? A before and after Covid-19 comparison. *Scientific Reports*, page (submitted).
